# Floodplain land cover affects biomass distribution of fish functional diversity in the Amazon River

**DOI:** 10.1038/s41598-019-52243-0

**Published:** 2019-11-13

**Authors:** Caroline C. Arantes, Kirk O. Winemiller, Alex Asher, Leandro Castello, Laura L. Hess, Miguel Petrere, Carlos E. C. Freitas

**Affiliations:** 1 Department of Wildlife and Fisheries Sciences, Texas A&M University, College Station, Texas, USA; 2 Present Address: Center for Global Change and Earth Observations, Michigan State University, East Lansing, USA; 3 Department of Statistics, Texas A&M University, College Station, Texas, USA; 4 Department of Fish and Wildlife Conservation, Virginia Polytechnic Institute and State University, Blacksburg, Virginia USA; 5 Earth Research Institute, University of California, Santa Barbara, California USA; 6 Programa de Pós-Graduação em Sustentabilidade de Ecossistemas Costeiros e Marinhos, UNISANTA, Santos, São Paulo Brazil; 7 Present Address: Programa de Pós-Graduação em Ecologia Aquática e Pesca, Universidade Federal do Pará, Belém, Pará Brazil; 8 Departamento de Ciências Pesqueiras, Universidade Federal do Amazonas, Manaus, Amazonas Brazil

**Keywords:** Community ecology, Conservation biology, Freshwater ecology, Tropical ecology, Wetlands ecology, Environmental impact

## Abstract

Land-cover change often shifts the distribution of biomass in animal communities. However, the effects of land-cover changes on functional diversity remain poorly understood for many organisms and ecosystems, particularly, for floodplains. We hypothesize that the biomass distribution of fish functional diversity in floodplains is associated with land cover, which would imply that fish traits affect behavioral and/or demographic responses to gradients of land cover. Using data from surveys of 462 habitats covering a range of land-cover conditions in the Amazon River floodplain, we fitted statistical models to explain landscape-scale variation in functional diversity and biomass of all fish species as well as subsets of species possessing different functional traits. Forest cover was positively associated with fish biomass and the strength of this relationship varied according to functional groups defined by life history, trophic, migration, and swimming-performance/microhabitat-use traits. Forty-two percent of the functional groups, including those inferred to have enhanced feeding opportunities, growth, and/or reproductive success within forested habitats, had greater biomass where forest cover was greater. Conversely, the biomass of other functional groups, including habitat generalists and those that directly exploit autochthonous food resources, did not vary significantly in relation to forest cover. The niche space occupied by local assemblages (functional richness) and dispersion in trait abundances (functional dispersion) tended to increase with forest cover. Our study supports the expectation that deforestation in the Amazon River floodplain affects not only fish biomass but also functional diversity, with some functional groups being particularly vulnerable.

## Introduction

Land-cover change is a major cause of degradation of floodplain ecosystems worldwide^1,2^, with expansion of cattle ranching and other kinds of agriculture leading to losses of biodiversity and biological productivity in tropical and subtropical regions^3^. Fish play key roles in ecological processes in tropical rivers and floodplains^4–6^, with several species supporting fisheries yields that provide income and food that sustain the livelihoods of millions of people^7^. Fish responses to land-cover change in floodplains should vary depending on functional traits^8^. Whereas land-cover changes can eliminate species with traits that are poorly adapted for the modified environment, changes may enhance fitness of other species that are able to take advantage of the new conditions, thus shifting the functional trait space occupied by the local assemblage^9^.

Tropical floodplain forests provide fishes with important food resources and seasonal access to critical nursery and refuge habitat^10,11^. In rivers with large and relatively unaltered floodplains, the biomass of several fish species derives from allochthonous food resources, including seeds, fruits, terrestrial insects as well as decaying forest vegetation^10–12^. Some fishes have morphology that facilitates efficient maneuvering within structurally complex habitats such as flooded tropical forests^8,13^. Fishes with other morphological and behavioral characteristics may have higher fitness in aquatic habitats associated with less forested floodplains. For example, species that feed on autochthonous resources, such as benthic algae or zooplankton, may benefit from higher aquatic primary production in areas lacking dense forest canopy^14^. Species possessing traits that enhance speed and/or efficiency of sustained swimming may be favored in relatively unstructured habitats^15^.

Analysis of functional trait diversity provides an effective means to assess community response to environmental change^9,16,17^, and form-function relationships have been established for a variety of taxa, with many showing predictable patterns of variation along environmental gradients^18,19^. Although some recent studies have examined effects of land-cover change on functional trait diversity in tropical streams^20–22^, these effects are poorly documented for tropical river-floodplain systems. To date, only three studies have assessed the effects of land-cover change on floodplain fishes or fishery yields, and these have produced mixed results regarding the role of functional traits in mediating shifts in the distribution of fish biomass. A recent study found that floodplain forest cover was positively correlated with total fishery yield for nine of the ten dominant taxa^23^. Another study found floodplain forest cover to be positively correlated with the biomass of every fish stock except for those of several detritivorous species^24^. Species composition of fish assemblages was found to vary across a gradient of forest cover in floodplains of the lower Amazon^8^. Based on analysis of beta diversity, that study suggested that positive responses to forest cover by ecological generalists might compensate for loss of specialists (e.g., species dependent upon structurally complex habitats), resulting in similar biomass in deforested and relatively undisturbed habitats^8^. However, no assessment has yet been made with regard to the distribution of fish biomass.

**Figure 1:**
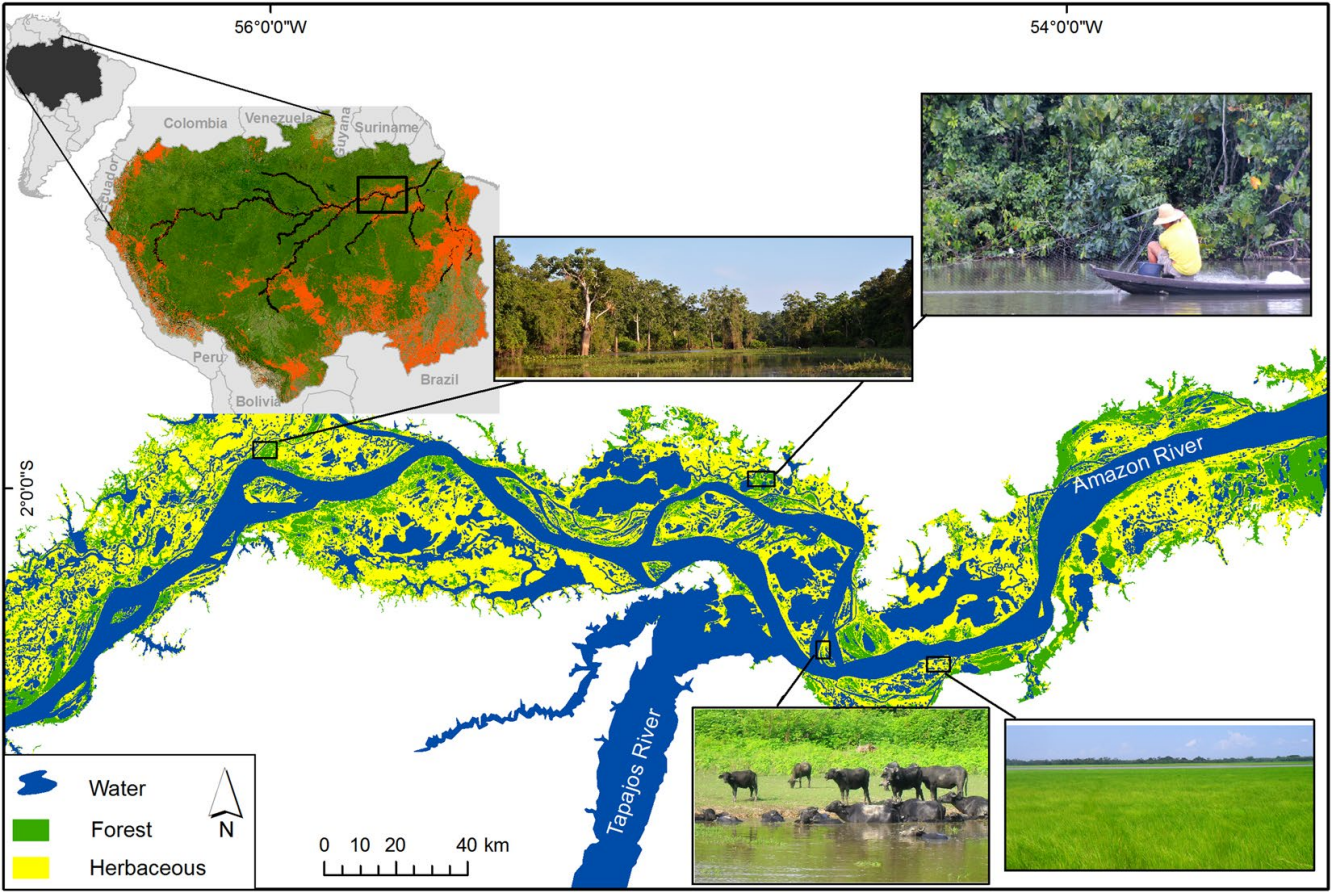
Study area in the lower Amazon floodplain showing land cover during the low-water period. Land-cover types are forest, herbaceous vegetation, and open water (lakes and secondary channels). In the lower Amazon region, vegetation consists primarily of herbaceous or shrub vegetation with only 13% forest cover^51^. Top left maps show the location of the Amazon Basin in South America (shaded black). Deforested areas within the basin are shaded in red^50,51,75^ and the study reach is enclosed in the rectangle. Photos (by L. Fernandes and C. C. Arantes): (**a**) forest surrounding a floodplain lake, (**b**) gillnet being set up for fish sampling, (**c**) water buffalo raised by local farmers, (**d**) floodplain area covered by herbaceous vegetation. Figure created in ArcGIS Desktop 10.6 http://desktop.arcgis.com/en/.

**Figure 2:**
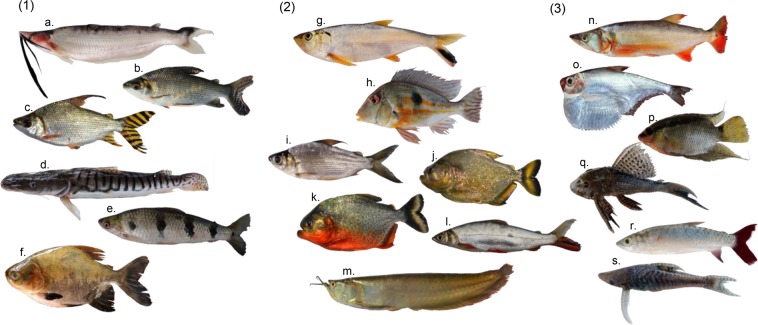
Examples of species possessing relatively high (1), medium (2), and low (3) importance for local fisheries (see Table S1). (1) (**a**) *Hypophthalmus fimbriatus*, (**b**) *Prochilodus nigricans*, (**c**) *Semaprochilodus insignis*, (**d**) *Pseudoplatystoma tigrinum*, (**e**) *Schizodon fasciatus*, (**f**) *Colossoma macropomum;* (2) (**g**) *Pellona castelnaeana*, (**h**) *Geophagus proximus*, (**i**) *Curimata inornata*,(**j**) *Serrasalmus maculatu*s, (**k**) *Pygocentrus nattereri*, (**l**) *Hemiodus microlepis*, (**m**) *Osteoglossum bicirrhosum*; (3) (**n**) *Acestrorhynchus abbreviatus*, (**o**) *Pristigaster cayana*, (**p**) *Mesonauta insignis*, (**q**) *Hypostomus plecostomus*, (**r**) *Chalceus epakros*, (**s**) *Hypoptopoma gulare*. Photos: C. C. Arantes, K. O. Winemiller, J. A. de Oliveira.

Here, we address two questions: Is the spatial distribution of fish biomass in the Amazon floodplain associated with land cover, and if so, can patterns be predicted from distributions of functional traits in local species assemblages? Answering these questions is critical for understanding species responses to land-cover change and for managing fisheries influenced by multiple anthropogenic stressors. We hypothesized that several traits representing life history, trophic, migration, and habitat-use strategies are related to land-cover gradients. We expected greater biomass of certain functional groups in habitats with greater forest cover (e.g., fish that occupy structurally complex habitats and fish that feed on allochthonous resources). Conversely, we expected the biomass of other groups (e.g., species with traits that facilitate foraging in open waters) to be less responsive to forest gradients. We further hypothesized that assemblage metrics are related to land-cover gradients. We expected the size of the niche space occupied by local assemblages (i.e., functional richness) and dispersion of traits within that space (i.e., functional dispersion) to increase with forest cover as a reflection of greater habitat complexity, resource diversity, and niche diversity in forested landscapes. To test these hypotheses, we surveyed fishes from diverse habitats and used satellite-mapped landscape data for floodplains of the lower Amazon River. Data were collected during four phases of the river’s annual hydrological cycle, and locations spanned a gradient of forest cover, from largely forested to almost completely deforested areas (Fig. 1). We modeled relationships between forest cover and total biomass of local fish assemblages as well as groups of species possessing different functional traits and degrees of importance for fisheries (Fig. 2, Tables 1 and 2). We also explored relationships between forest cover and functional diversity metrics that have been used increasingly to infer community assembly processes and responses to environmental variation^9,25,26^. Our findings reveal the potential vulnerability of fish stocks, fishery production, and functional diversity to forest loss.

**Table 1:** Summary of estimates (lower quartile (LQ), median, upper quartile (UQ)) and descriptions of methods and variables for floodplain land cover and local environmental features in lake systems of the lower Amazon floodplain based on 462 habitats surveyed during four stages of the annual hydrological cycle.

Variable	Description or method	LQ	Median	UQ
***Land-cover category within the lake system***				
Forest (%)	Percent of closed-canopy tree cover and short trees, shrub, or semi-shrub (including the aroid *Montrichardia arborescens*) in the lake system based on satellite imagery.	10.5	21.0	47.6
Open water (%)	Percent of open water in lakes and channels during low waters in the lake system (excluding the mainstem Amazon channel) based on satellite imagery.	3.0	9.6	12.9
Herbaceous vegetation (%)	Percent of grasses, forbs, soil, or fresh sediments during low waters in the lake system based on satellite imagery.	42.6	66.9	74.5
Macrophyte - geoprocessing: Macro (geop)	“Macro (geop)” index indicating the percent of the lake system with macrophytes present (during late December to January) in three or more of 5 years analyzed (2006/2007 to 2010/2011) based on ALOS PALSAR satellite imagery (Supplementary Methods).	13	17.1	22.5
***Local environmental variables (PC1 and PC2)***				
Macrophyte - visual observation (%): Macro (obs)	Percent coverage of water body by macrophytes as estimated through visual inspection of the habitat. “Macro (obs)” matches the scale and sampling dates of our local fish assemblage surveys.	3.0	10.0	40.0
Depth (m)	Averages based on measurements in various locations within each habitat.	1.5	2.1	3.1
Dissolved oxygen (mg/l)		1.6	2.2	3.4
Transparency (cm)		30.0	44.5	60.0
Temperature (°C)		29.3	30.1	30.8
***Season category***				
Low water, rising water, high water and falling water	River water levels in the lower Amazon begin to rise during December and reach a maximum during late May or early June. The water level starts to fall during August, reaching its minimum during November. Low water levels reduce aquatic habitats and their connectivity, and high-water levels greatly expand the flooded area and aquatic habitat.			
***Floodplain habitat type category***				
Lake (open water)	Floodplain depressions that normally hold water throughout the flood cycle.			
Secondary channel (open water)	Channels transporting river waters across sections of floodplains.			
Flooded forest	Riparian forests are inundated for 1–5 mo/yr, depending on elevation of the terrain. Food is generally abundant, and many fishes feed on plants, detritus, or invertebrates in newly flooded areas.			
Flooded herbaceous (campo)	Seasonal grasses or forbs, or sparse and short shrubs that are inundated for about six to nine months per year, depending on terrain elevation. Most areas transition to open water or aquatic macrophyte at high-water stage and may be bare when newly exposed by falling water levels.			
***Management***	Classified as present or absent based on interviews with local fishers and community leaders. Management was considered to be present when there were restrictions on fishing gear, species, location or seasons.			

PCA was used to ordinate habitats according to gradients defined by local environmental variables (see Methods).

**Table 2:** Functional traits categories analyzed in this study. Numbers in parentheses represent the number of species within each group.

Functional group category	Functional group	Description
Trophic strategies	Herbivores (18)	Feed predominantly on C3 or C4 plant material (seeds, fruits or leaves) and on filamentous algae.
	Omnivores (47)	Ingest combinations of plant material, detritus, and invertebrates.
	Detritivores (28)	Predominantly ingest fine particulate organic matter and non-living macrophyte tissues, but also on filamentous algae.
	Invertivores (23)	Ingest variable fractions of aquatic and terrestrial insects, microcrustaceans from the benthos or water column, spiders, shrimps, and mollusks.
	Planktivores (10)	Ingest phytoplankton, zooplankton, and occasionally small amounts of plant material and detritus.
	Piscivores (45)	Ingest adult, juvenile, or larval fish, either whole or in pieces, including scales and fins.
	Piscivores-macroinvertivores (14)	Feed on the same sources as piscivores but also ingest significant fractions of diverse terrestrial or aquatic macroinvertebrates (e.g., Ephemeroptera, Chironomidae, Coleoptera, Crustacea, etc.).
Migratory behaviors	Sedentary (55)	Resident species that spend their entire life-cycles within floodplain habitats eventually performing short-distance movements. Sedentary species were small-bodied species, or had territorial behavior, or are known to be strongly associated with substrates or complex structured habitat (e.g., tree branches and aquatic vegetation).
	Local migrators (120)	Diverse group of fishes that migrate laterally from floodplain lakes or river channels onto flooded floodplain habitats following closely the dynamic ‘pulsing’ of water levels
	Regional migrators (8)	Species that migrate onto flooded floodplains habitats during high waters, but also conduct longitudinal migrations (often hundreds of kilometers) along river channels to spawn, particularly during falling waters
	Long-distance migrators (3)	Species that migrate thousands of kilometers along river channels, though their juveniles often inhabit floodplain lakes
Life history strategies	Equilibrium with maturation at small sizes (16)	Maturation at small size (<120 mm standard length, SL), low batch fecundity, large oocytes, well-developed parental care, and maximum body size between 97–269 mm SL.
	Equilibrium strategists with maturation at large size (16)	Maturation at large size (>170 mm SL), low batch fecundity, large oocytes, well-developed parental care and maximum size > 400 mm SL.
	Periodic strategists with maturation at small size (73)	Maturation at small size (between 63–148 mm SL), varied batch fecundity size (average ~ 4,000), small oocytes, maximum size between 137–410 mm SL and no parental care.
	Periodic strategists with maturation at large size (43)	Maturation at large size (>164 mm SL), batch fecundity highly variable, small oocytes, no parental care and maximum size > 253 mm SL.
	Intermediate strategists (32)	Batch fecundity between 1,000 and 9,000, relatively large oocytes, and intermediate development of parental care.
	Opportunistic (5)	Small size (between 26–113 mm SL), early maturation (<60 mm SL), high and sustained reproductive effort but low batch fecundity and no parental care.
Swimming behavior/microhabitat use	Nektonic maneuverable (41)	Laterally compressed body and superior mouth position. Morphological traits associated with efficient swimming performance based on a hydrodynamic body and feeding within the water column.
	Nektonic burst swimmers (18)	Fusiform body and terminal mouth position. Morphological traits associated with efficient swimming performance based on a hydrodynamic body and feeding within the water column.
	Surface dwellers (2)	Intermediate lateral body compression, superior mouth and either deep or fusiform body. More dorsally than laterally positioned eyes.
	Epibenthic maneuverable (57)	Relatively deep body that is less hydrodynamic than nektonic maneuverable fishes but efficient in making lateral and vertical turns. More dorsally than laterally positioned eyes.
	Benthic-slow (36)	Relatively wide body, dorsally located eyes, and inferior mouth, which are characteristic of bottom dwellers. Low muscle mass and pectoral and caudal fin areas. Three species (e.g., *Hoplias malabaricus)* had terminal or superior mouths.
	Benthic-fast (23)	Relatively wide body, dorsally located eyes, and inferior mouth, which are characteristic of bottom dwellers. Higher muscle mass and caudal fin aspect ratio – traits associated with more efficient sustained swimming compared to benthic-slow fishes.
	Gymnotiforms (8)	Diverse group of electric fishes that move using undulatory motion of the anal fin, either substrate or aquatic vegetation dwellers, inactive during daylight but actively forage during the night using weak electric organ to locate prey.

## Results

**Figure 3:**
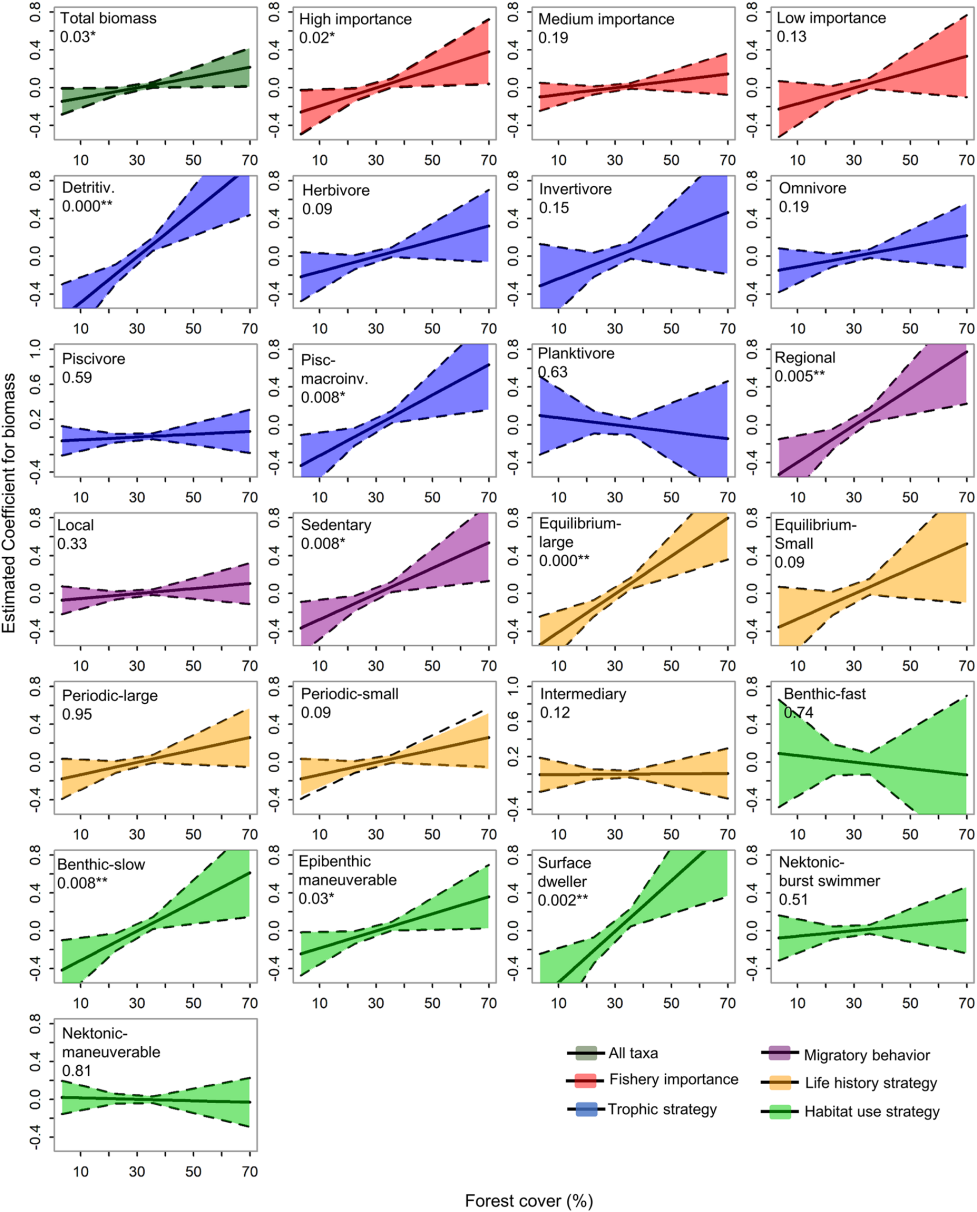
Estimated regression coefficient (partial effects) of forest cover on the relative biomass of fish (CPUE) for each fish group. Steepness of the slopes indicates the strength of the relationship with forest cover. Confidence intervals (95%) are shaded in color; functional groups’ names and P values are indicated; *p ≤ 0.05; **p ≤ 0.008 (significant after Bonferroni correction; see details in methods).

Total fish biomass and biomass of several functional groups (42%) were positively associated with forest cover, and the strength of these relationships depended on the traits possessed by each group. Habitats within catchments with greater forest cover tended to have greater total fish biomass (p = 0.03) and biomass of species that are important for commercial fisheries (p = 0.02). Biomass of detritivores and equilibrium strategists that mature at large sizes was positively and even more strongly associated with forest cover (p < 0.0001; Fig. 3). Piscivore-macroinvertivores, sedentary species, regional migrators, and surface dwellers also had greater biomass in catchments with more forest cover (all p < 0.001). Forest cover also was associated with biomass of benthic-slow (p = 0.008) and epibenthic-maneuverable species (p = 0.003).

**Figure 4:**
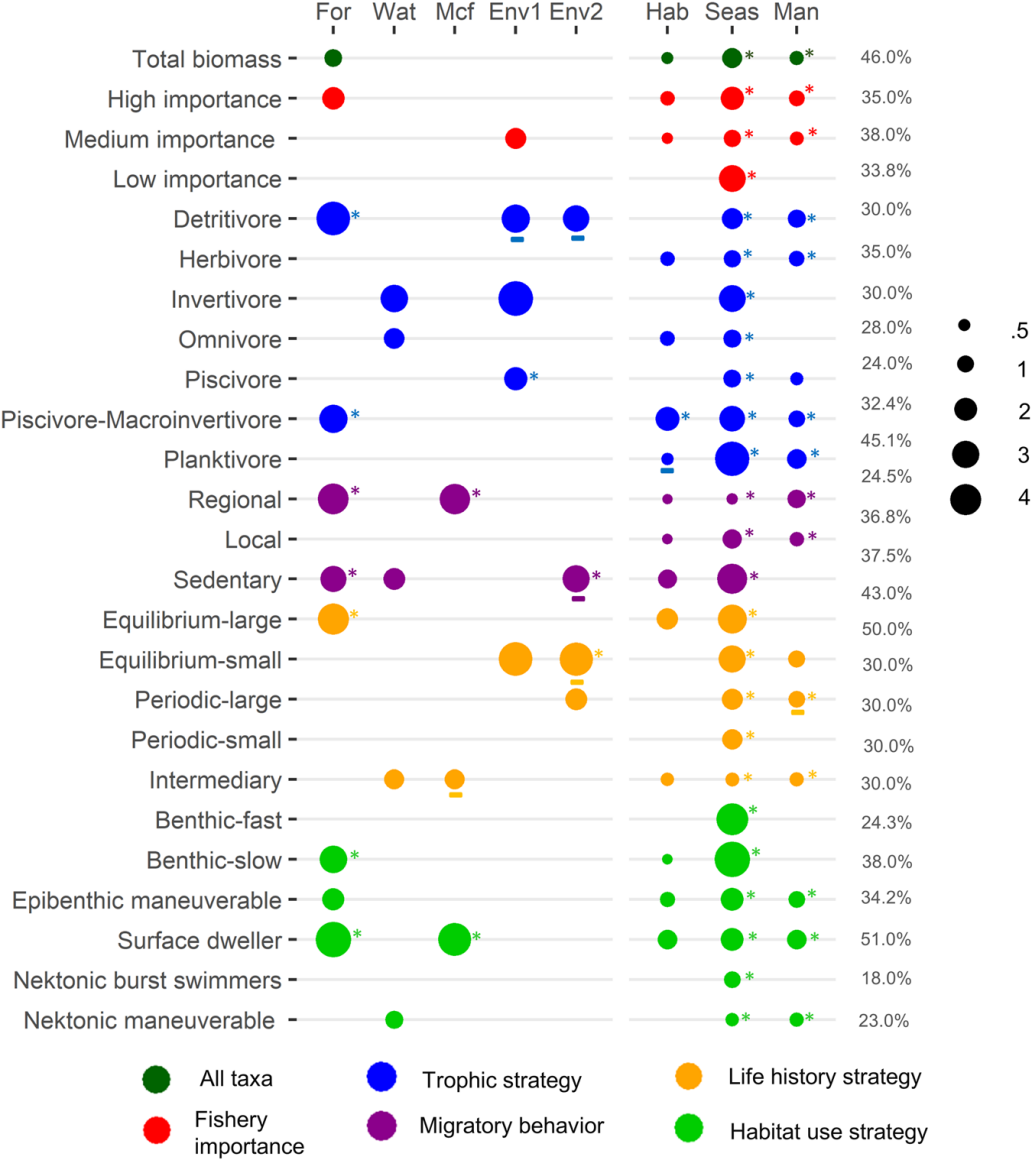
Regression coefficients for CPUE of total fish biomass (all taxa), groups of species possessing different degrees of importance for fisheries (high importance, medium importance and low importance) and different functional traits (groups of trophic, migration, life history, and swimming/microhabitat-use strategies) as a function of forest cover (For), open water (Wat), large-scale (Macro (geop)) estimate of aquatic macrophyte cover (Mcf), local environmental variables (reduced as PCA1 (Env1) and PCA2 (Env2)), habitat type (Hab), season (Seas), and presence of management (Man). Circle size represents the relative contribution of predictors, shown by standardized coefficients. Lines below the coefficients indicate negative effects. Coefficients are presented only for relations that were significant at p ≤ 0.05 and relationships that were significant at p < 0.008 (Bonferroni corrections) are highlighted by asterisks (*). Deviance explained (%) is presented for regression models (right column).

The biomass of several other groups (28% of all groups) tended to be greater in lake systems with greater forest cover, but these relationships were not statistically significant (p > 0.09) (Fig. 3). These groups were invertivores, herbivores, omnivores, equilibrium strategists with maturation at small size, periodic strategists with maturation at small size, periodic strategists that mature at large size, and species of low importance for fisheries. Biomass of the remaining 32% of the fish groups did not show any relation with forest cover (Figs 3 and 4). These groups were piscivores, planktivores, species with an intermediate life history strategy, local migrators, species classified as having benthic-fast, nektonic-burst, or nektonic-maneuverable swimming behavior, and species of moderate importance for fisheries (Figs 3 and 4).

In addition to forest cover, several other variables were correlated with the biomass of various fish groups. Most groups (58%) had significantly greater biomass in areas where management was present (Figs 4, S2). Fish biomass also was influenced by the floodplain habitat category. When the effects of other variables were controlled statistically, biomass of most (80%) groups was greatest in areas of flooded forest when compared to other habitats (Fig. S3). Fish biomass also was influenced by hydrology (Fig. 4). Again, after controlling for other variables, the strongest relationships between fish biomass and forest cover were generally observed during the low-water season, followed by the falling-water period (Fig. S4). Few functional groups had significant relationships with other land-cover variables (open water 19% of groups, macrophytes 12% of groups) or with local environmental factors (PC1 19% of groups, PC2 15% of groups).

Functional richness and dispersion both were positively associated with forest cover (p < 0.001). Functional richness also showed significant relationships with local environmental variables (reduced as PCA1 and PCA2), presence of management, habitat type and season, whereas functional dispersion was associated with aquatic macrophyte cover (Macro (geop), local environmental variables (reduced as PCA1), habitat type, and season (Table 3). Models explained 53% and 33% of the variations in functional richness and dispersion, respectively.

**Table 3:** Coefficients estimates, standard errors (SE) and p-values of the relationships between functional richness and dispersion and the floodplain land cover, local environmental variables, habitat types, season categories and the presence of management.

	Functional richness			Functional dispersion		
	Estimate	SE	P*-value*	Estimate	SE	P-*value*
Intercept	1.482	0.100	<0.001	0.211	0.023	<0.001
**Land-cover category**						
Forest (%)	0.003	0.001	0.01	0.001	0.000	<0.001
Open water (%)	0.005	0.003	0.14	0.001	0.001	0.21
Macro (geop)	0.004	0.002	0.07	0.001	0.001	0.02
**Local environmental variables**						
PC1	0.311	0.090	<0.001	0.022	0.021	0.29
PC2	−0.186	0.074	0.01	−0.046	0.017	0.01
**Season category**						
Low water	0.827	0.093	<0.001	0.109	0.022	<0.001
Rising water	0.134	0.048	0.01	0.003	0.011	0.78
High water						
Falling water	0.381	0.058	<0.001	0.027	0.013	0.04
**Floodplain habitat type**						
Lake	−0.233	0.054	<0.001	−0.062	0.012	<0.001
Secondary channel	−0.194	0.057	<0.001	−0.046	0.013	<0.001
Flooded forest	0.117	0.056	0.04	0.044	0.013	<0.001
Flooded herbaceous (campo)						
**Management**	0.081	0.040	0.05	0.002	0.009	0.84

Reference levels for season were ‘high water’ and for habitat type ‘Flooded herbaceous (campo)’.

Plots of randomized quantile residuals and residuals and fitted-versus-residual showed that the residuals were normally distributed with no apparent trends, indicating that the models had good fit (Fig. S5). Values for Moran’s I did not differ from random expectations, indicating no significant spatial dependence in the data (Fig. S6).

## Discussion

Although our results do not provide conclusive evidence, e.g., by directly comparing fish biomass before and after land-cover change, they nonetheless provide indirect evidence that forest loss negatively impacts fish biomass and assemblage functional diversity at local and regional scales in floodplains of the lower Amazon. In agreement with our hypotheses, the biomass of several functional groups (42%) was positively associated with forest cover. These groups include detritivores, equilibrium strategists with maturation at large size, piscivore-macroinvertivores, surface dwellers, benthic-slow, sedentary, and epibenthic-maneuverable species, as well as species that undergo regional migrations. Biomass of other functional groups appear to be unrelated to forest cover, including species possessing good dispersal capability and some that are considered habitat generalists. Despite these differential responses of functional groups to landscape gradients, the total fish biomass and the biomass of species with high fisheries importance as well as functional diversity metrics tended to increase with forest cover, suggesting that possible niche complementarity may facilitate species coexistence in habitats of forested areas. If indeed functional traits affect fish biomass dynamics in response to land cover, functional traits may provide an effective means to predict future compositional shifts in fish assemblages as floodplains change in response to natural and anthropogenic processes.

Differential responses of functional groups to forest cover is consistent with the view that trait-mediated environmental filtering drives population/community responses to environmental and anthropogenic gradients^20^. Consistent with previous studies showing that certain traits become less prevalent or lost from species assemblages during landscape transformation (‘performance filtering hypothesis’^9,27,28^, our analysis of the biomass distribution of fish functional groups along land-cover gradients indicates that certain species and traits are particularly vulnerable to forest loss. Similarly, results showing positive relations of functional richness (number of unique trait combinations) and trait dispersion (relative abundance within trait space) with gradients of forest cover suggest that environmental changes associated with forest loss may filter out certain species and traits. In the Amazon, many fish species are known to exploit flooded forests where they have enhanced feeding opportunities, growth rates, and/or reproductive success. Detritivores may select forested areas that contain detritus of greater nutritional value (e.g., high levels of amino acids)^11,29^. Migratory prochilodontids and sedentary-benthic fishes, such as loricariid catfishes, feed on organic matter derived from decomposed forest vegetation that contains fungi and bacteria of high nutritional value^30^. Fishes with equilibrium life history strategies, such as the mouth-brooding aruana (*Osteoglossum bicirrhosum*) and cichlids with bi-parental brood guarding, may have enhanced offspring survival and recruitment within structurally complex habitats of flooded forests that provide cover from predators. Several fishes that migrate longitudinally along river channels (i.e, regional migrators such as *Prochilodus nigricans*, *Semaprochilodus* spp., *Brycon* spp., *Colossoma macropomum*) return to floodplains during high-water periods and likely enter flooded forests for refuge and feeding opportunities^11,15,31^. Other fishes consume fruits and seeds (e.g., *Piaractus brachypomus*) or insects (e.g., *O*. *bicirrhosum*) that fall into the water. Epibenthic maneuverable fishes, such as cichlids, are well adapted to forage and evade predators within the structurally complex habitats of flooded forests^32^. Conversely, other functional groups, such as nektonic piscivores and planktivores, were not significantly associated with forest cover, possibly because these groups can more effectively exploit resources in habitats associated with other land-cover categories (e.g., open water, herbaceous vegetation). Biomass of planktivorous fishes was weakly negatively (but not statistically significantly) correlated with forest cover, possibly because dense forest canopies limit light to support phytoplankton production, which in turn would limit zooplankton abundance. Alternatively, certain land uses associated with low forest cover, such as pasture for livestock and fields for crops, might increase inputs of inorganic nutrients into aquatic systems^33^, thereby increasing primary productivity and the biomass of planktivores. These examples further indicate that shifts in environmental conditions associated with forest loss favor some functional groups but are detrimental to others. Deeper knowledge on how forest cover influences fish stocks could be gained from comparative or experimental research that analyzes not only functional traits (e.g., those directly affecting food acquisition, growth, survival and reproduction), but also performance measures (e.g., metabolic rate, growth rate, recruitment) that determine the spatial and temporal distribution of biomass.

Certain functional groups (e.g., herbivores, invertivores, local migrators) were positively correlated with forest cover (see trends in Fig. 3), but those relationships were weak and not statistically significant. This result is possibly due to sampling constraints or because functional groups were defined too broadly, thus obscuring key determinants of spatial abundance patterns. Although our study employed extensive spatial and temporal sampling, greater sampling effort within local habitats might reveal stronger patterns for biomass of herbivores, invertivores, equilibrium strategists with maturation at small size, and periodic strategists, which in our study were positively but not significantly associated with forest cover. Some of these fishes likely exploit food resources within flooded forest (e.g., several piranha species (Serrasalmidae) feed on fruits and seeds)^6,11^, but also may have sufficiently generalized niches to allow exploitation of resources in non-forested areas. Biomass of other fish groups (e.g, local migrators, species of medium importance for fisheries) had no relationship with forest cover, and many of the species in these groups apparently are ecological generalists. For example, the group “local migrators” comprised more than a hundred species, including carnivorous piranhas that have broad diets and high abundance in diverse habitats (e.g., *Pygocentrus nattereri*, *Serrassalmus* spp.). If we exclude these ecological generalists from the analysis, the biomass of local migrators was significantly greater where there was more forest cover. Stronger patterns might be revealed not only by increasing sampling effort, but also by including additional traits and using statistical methods that reduce redundancy and multidimensionality in functional trait datasets and produce continuous measures of functional diversity^34^.

Although not the focus of our study, spatial variation in functional diversity could affect ecosystems processes in the Amazon floodplain^16,35^. Most detritivorous fishes (e.g., curimatids, prochilodontids, loricariids) had positive relationships with forest cover, and some of these fishes have been shown to play important roles in sediment and nutrient dynamics with effects on benthic invertebrates^4,5,36,37^. Obviously, more work is needed not only to understand the functional roles of species and functional groups, but also to develop our understanding of the environmental filters that are involved in land-use change in floodplains and the consequences of losing functional diversity as a consequence of human activities in the Amazon.

Seasonal hydrology, local habitat conditions, and fisheries management also influenced fish biomass and functional diversity. In our study, the biomass of all functional groups was strongly associated with seasonal hydrology, a finding consistent with previous studies that concluded hydrology is the major driver of fisheries production and assemblage dynamics in the Amazon floodplains^38,39^. Most Amazon fishes, at a minimum, can undergo local-scale movements during various phases of the annual flood pulse. Given this potential for dispersal and habitat selection, it might be expected that biomass of most functional groups is associated with local habitat conditions. During the height of the flood pulse, many fishes inhabit submerged forested areas; during floodwater recession, these fishes are forced to migrate into channels or lakes^40–42^. The great expansion of aquatic habitat during the flood pulse results in lower catch rates in gillnets, the collecting gear employed in our study. Despite the fact that sampling should be less efficient during the high-water phase, most functional groups had greater biomass in flooded forest habitats when compared with the other habitats, supporting the inference that many Amazonian fishes have evolved a strong dependence on conditions and resources provided by flooded forests^11^.

Our findings indicate that fishery management at the local scale enhances biomass and functional richness of diverse species, including those with the greatest economic importance. Biomass of several functional groups, including species important for fisheries, was greater at locations with effective fisheries management. A previous study showed how Amazonian fishing communities that implemented and enforced fishing regulations had nearly 50% more stock abundance compared to those without management^43^. A general lack of effective fisheries management in the Amazon, including failure to enforce restrictions for fishing methods, seasons and catches, has impacted the distribution of fish biomass and functional structure of local assemblages.

The biomass of a few functional groups was significantly correlated with land cover variables other than forest cover and with variables describing local environmental conditions. However, for most groups, these explanatory variables were less important than forest cover. Biomass of surface-dwelling fishes was positively correlated with aquatic macrophyte cover. Invertivore biomass was positively correlated with water transparency. Biomass of highly-maneuverable nektonic fishes and those with intermediate life-history strategists were positively correlated with open-water cover. Yet considerable variance remained unexplained for most groups. Given the vastness, heterogeneity, and dynamic nature of the Amazon floodplain, it seems unlikely that spatial distributions of fish biomass and patterns of functional diversity as well as mechanisms driving such patterns can be revealed by a single study regardless of spatial and temporal extent. Because fishes and many other aquatic organisms disperse in response to seasonal hydrology, várzea metacommunities are seasonally dynamic^44^. In addition, várzea fishes have evolved ecological strategies that allow them to exploit changing environmental conditions in the floodplain mosaic (e.g., flexible feeding behavior and/or compensatory responses in growth or fecundity)^45,46^. Further understanding of the mechanisms driving spatial patterns of fish biomass in floodplains could be achieved by including additional geospatial variables relevant for fishes, such as estimates of habitat connectivity during various phases of the annual hydrologic cycle.

Amazonian fishes have evolved for tens of millions of years in pulsing fluvial systems surrounded by forest^47^, and many of them are adapted to exploit resources and conditions within flooded forests^11^. Our study supports the expectation that floodplain degradation, including deforestation and disruption of natural flow regimes, will reduce fish biomass and functional diversity, with some functional groups being particularly vulnerable to changes. These groups include species that constitute major conduits of matter and energy in food webs^31,48^, influence nutrient cycling^4,37^, and sustain important fisheries^49^. Finally, we propose that functional trait sets could be used to predict changes in the distribution of fish biomass after changes in land use and other anthropogenic impacts.

## Methods

### Study area and data collection

The study was conducted in the floodplain of the lower Amazon River (referred to locally as *várzea*) in an area of 17,674 km² in Brazil (Fig. 1). The study area contains a mosaic of forests and herbaceous vegetation, lakes, and secondary channels. The annual flood pulse is monomodal, and water level varies 5.7 m on average. Nearly the entire floodplain is covered with water during high-water periods, and lakes and connecting channels retain water after the floodplain drains during low-water periods. Over the past 40 years, large areas of várzea in the lower Amazon were deforested for agriculture^50^. Jute (*Corchorus capsularis*) plantations and cattle ranching resulted in a loss of 56% of floodplain forest cover by 2008^50,51^. Over the past 30 years, 78% of the deforested area was replaced with herbaceous vegetation, 5% is bare soil where ground cover has not yet regenerated, and 16% contains open water^51^.

Floodplain forest was mapped at 30-m resolution using Landsat Thematic Mapper images (see methods details in Table 1 and Supplementary Material and Methods), and data obtained from this remotely sensed imagery were assembled according to spatial units defined as local catchments (or *“lake systems”* sensu^8,23^). The 20 lake systems (Fig. 1, median area: 23.4 km^2^) encompassed a gradient of forest cover ranging from 3 to 70%. For the same lake systems, we measured three additional land-cover variables (percent cover of open water, herbaceous vegetation and macrophyte within lake systems) that along with forest represent the principal land-cover types available for fishes within the floodplain (see Table 1 for descriptions and Fig. 1 for images).

Fish biomass data were obtained from standardized fish surveys conducted in 462 floodplain habitats within these 20 lake systems distributed approximately 250 km along the lower Amazon River (Fig. 1). For each habitat type within each lake system, and during dry, rising-, high-, and falling-water periods, we collected fish using a standard set of nets with different mesh sizes (11 gillnets measuring 25 × 2 meters, with mesh sizes 20, 30, 40, 50, 60, 70, 80, 90, 100, 120, and 130 mm, and one gillnet measuring 100 × 3 meters, with 180 mm mesh) to catch multiple fish size classes and species (study area and detailed survey descriptions are presented in the Supplementary Material and Methods). Biomass data were standardized as catch-per-unit effort (CPUE = biomass of fishes caught divided by hours of net in water). All applicable institutional and national guidelines for the care and use of animals were followed.

(Approval of Animal Use Protocol- Texas A&M University IACUC 2013-0099, Reference Number: 004728 and Ministério do Meio Ambiente - MMA, Instituto Chico Mendes de Conservação da Biodiversidade - ICMBio, Sistema de Autorização e Informação em Biodiversidade - SISBIO-Brazil. Reference Number: 30852-5).

During each season within each habitat of each lake system where we collected fish, we measured water temperature, dissolved oxygen concentration, depth and transparency (Table 1). We visually estimated the area covered by aquatic macrophytes (Table 1). Macrophyte indices obtained from remotely sensed imagery provided large-scale estimates of aquatic plant coverage in lake systems, and visual estimates were made during fish surveys to characterize aquatic plant coverage at a local scale. Finally, we classified the habitats according to the presence or absence of management practices based on interviews with local fishers (Table 1).

### Traits classification and data analyses

We used statistical modelling to test whether greater forest cover is related to greater biomass of fishes, both collectively within local habitats, and for groups of species possessing different functional traits and degrees of importance for local fisheries. We also used models to directly explore relationships between assemblage functional structure and forest cover gradients.

Species were grouped according to their degree of importance in local fisheries, and this classification was based on their relative contribution to total yields landed in the main cities in the lower Amazon^39^ (Fig. 2). Fish of *high importance* (28 species) contributed ≥85% of the total landing. Fish of *medium importance* (83 species) contributed ≤15% of the total landing. Seventy-four species were classified as having *low importance* and were rarely landed for sale as food, although some of them are used as bait or sold as ornamental fish (Fig. 2). The other categories comprised functional groups based on trophic, migration, life history, and swimming behavior/microhabitat-use strategies (Table 2). We classified species according to eight trophic and four migration strategies based on information on diets and dispersal behavior, respectively, from published reports. Migratory strategies of Amazon fish often are related to reproduction and/or feeding ecology and influenced by seasonal hydrology and physical-chemical conditions of habitats in the riverscape. We classified species according to six life history strategies based on maximum body size, size at maturation, batch fecundity, and parental investment per individual offspring following refs^52–54^. Finally, we classified species according to five strategies of swimming behavior/microhabitat use based on the classification of ref.^8^ that uses traits associated with swimming performance and vertical position within the water column during foraging, phenotypes that influence fitness along gradients of habitat structural complexity and other environmental features^18,55^. Due to their small sample sizes, species belonging to groups associated with an opportunistic life history strategy, long-distance migration, or gymnotiform swimming mode were not included in the analyses. Detailed descriptions of species classifications and references can be found in Table 2, Supplementary Methods, Supplementary References and Table S1.

Our analyses also accounted for seasonality, habitat type, presence/absence of local fisheries management, local environmental conditions, and three additional land-cover variables (percent cover of open water, herbaceous vegetation, and aquatic macrophytes within lake systems) that along with forest represent the principal land-cover categories within the floodplain (see Table 1 for descriptions and Fig. 1 for images); these variables have previously been reported to influence fish composition and fisheries yields in the Amazon floodplain^8,39,56^. We excluded herbaceous vegetation cover from analyses because it was highly correlated with forest cover (r = −0.96), and we therefore assumed that response variables (multispecies CPUE, CPUE of groups, functional diversity) that were positively related to forest cover were inversely associated with herbaceous cover. Correlations among other independent variables were <0.4, including correlation between forest cover and presence or absence of management (r = 0.15). The five local environmental variables were reduced to two variables by using scores on the first two axes from principal components analysis (PCA) (Fig. S1). PC1 described a gradient principally influenced by water transparency and dissolved oxygen concentration, and PC2 modeled a gradient of macrophyte cover, water temperature, and depth.

To test whether greater forest cover is related to greater biomass of fishes, we modeled total fish biomass (CPUE) within local habitats and biomass of groups of species having different functional traits and degrees of importance for fisheries as a function of linear predictors (Tables 1 and 2). We fitted generalized linear models (GLM) assuming a Poisson-Gamma distribution from the family Tweedie, the set of exponential distributions indexed by a power parameter^57,58^. Frequent zero catches, such as observed for our CPUE data, is a common issue in fishery modeling that is addressed in a straightforward manner by this method^57,58^. This distribution handles zero values uniformly with positive and continuous values, and it was found to outperform other models used for CPUE data containing many zeros (e.g., delta models, generalized linear models with an additive constant)^59–61^ (see Supplementary Material). We used Bonferroni adjustment to set the statistical significance level based on multiple comparisons (Bonferroni correction: *p*_i_ ≤ α/*m*, where *m*_0_ is the number of null hypotheses, in our case six corresponding to the fish groups described above (Tables 2 and S1); yielding *p*_i_ = 0.008). The use of Bonferroni correction requires caution because, although the method reduces the chances of type I error (concluding that a significant difference is present when it is not), it increases the chances of type II error such that real differences may not be detected^62,63^. To avoid such errors and facilitate critical interpretation of our statistical results, we therefore present actual p-values and highlight those <0.008 (Fig. 3). Model fit and assumptions were judged by visual inspection of randomized quantile residuals^64^.

To test whether functional assemblage structure is related to land-cover gradients, we also used continuous measures of functional diversity that use quantitative values for functional traits. We calculated two functional diversity measures: functional richness and functional dispersion^25,65^. Functional richness represents the functional space occupied by the assemblage. Because our functional traits were defined as categorical variables, functional richness was calculated as the number of unique trait combinations. Functional dispersion is the mean distance of individual species to the centroid of all species in the community^65^. To estimate functional dispersion, we used principal coordinates analysis (PCoA) axes as the new quantitative traits^25^. PCoA axes were computed from a Gower dissimilarity matrix among species^65^. Then, functional dispersion was calculated from the mean distance of species to the centroid of the resulting multivariate trait space with each distance weighted by the relative abundance of the corresponding species^65^. We used GLM assuming a Gaussian distribution to test relationships between these functional diversity metrics and forest cover and the other linear predictors (Table 1). Model diagnostics were checked by visually inspecting autocorrelation of residuals and fitted-versus-residual plots.

Moran’s I statistic was used to evaluate whether there was significant spatial dependence in the data that was not captured by the models^66,67^ (see Supplementary Methods). Analyses were performed in R v.3.3.3. Models were fitted using the *statmod*^68^, *Tweedie*^69^, and *stats*^70^ packages. Moran’s I was calculated using the *ape*^71^, *geoR*^72^ and *fields*^73^ packages. Functional diversity metrics were calculated using the *FD* package^65,74^.

## Supplementary information

Supplementary Information

## Data Availability

Data will be available in a repository platform such as dryad (https://datadryad.org/).
