# Supplementary material for: Floodplain land cover affects biomass distribution of fish functional diversity in the Amazon River
Source: Sci Rep. 2019 Nov 13;9:16684. doi: 10.1038/s41598-019-52243-0 (PMC6853970; doi:10.1038/s41598-019-52243-0)
Supplement: Supplementary file 1 — Supplementary Information [file 41598_2019_52243_MOESM1_ESM.pdf]

Floodplain land cover affects biomass distribution of fish functional diversity in the Amazon River

Authors: Caroline C. Arantes[1,2*], Kirk O. Winemiller[1], Alex Asher[3], Leandro Castello[4], Laura L. Hess[5], Miguel Petrere Jr.[6,7], Carlos E. C. Freitas[8]

[1]Department of Wildlife and Fisheries Sciences, Texas A&M University, College Station, Texas, USA.

[2]Present address: Center for Global Change and Earth Observations, Michigan State University, East Lansing, USA.

carolinearan@gmail.com

[3]Department of Statistics, Texas A&M University, College Station, Texas, USA.

[4]Department of Fish and Wildlife Conservation, Virginia Polytechnic Institute and State University, Blacksburg, Virginia, USA.

[5]Earth Research Institute, University of California, Santa Barbara, California, USA.

[6]Programa de Pós-Graduação em Sustentabilidade de Ecossistemas Costeiros e Marinhos, UNISANTA, Santos, Sao Paulo, Brazil.

[7]Present address: Programa de Pós-Graduação em Ecologia Aquática e Pesca, Universidade Federal do Pará, Belém, Pará, Brazil.

[8]Departamento de Ciências Pesqueiras, Universidade Federal do Amazonas, Manaus, Amazonas, Brazil.

Supplementary Information

Supplementary Methods - Detailed descriptions of study area, data collection, species classification, and statistical analyses

Study area

The study was conducted in the floodplain of the lower Amazon River (referred to locally as *várzea*) in an area of 17,674 km² in Pará State, Brazil (Fig. 1). The study area contains a mosaic of forests, lakes, and secondary channels that are continually re-shaped by dynamic processes of erosion and sedimentation (Junk 1997). Várzea supports diverse plant communities, with herbaceous and shrub communities occurring near margins of lakes and channels, and forests occupying higher ground along natural levees (Junk et al. 2012). The annual river flood pulse as measured at the Óbidos gauge (ANA 2014) is monomodal and varies 4 to 8 m annually (mean of 5.7 m), creating marked differences in floodplain conditions between high-water periods, when most areas are flooded, and low-water periods when only lakes and connecting channels retain water. The river water level begins to rise during December and reaches its maximum during late May or early June. The water level starts to fall during August, reaching its minimum during November. Historically, large areas of várzea in the lower Amazon were deforested for agriculture (Sheikh et al. 2006, WinklerPrins 2006). Jute (*Corchorus capsularis*) plantations and cattle ranching were the dominant agricultural activities during the 20th century, leading to a loss of 56% of floodplain forest cover by 2008 (Renó et al. 2011). Over the past 30 years, 78% of the deforested area was replaced with herbaceous vegetation, 5% is bare soil where ground cover has not yet regenerated, and 16% contains open water (Renó et al. 2011).

Satellite imagery

Floodplain land cover classes mapped were: open water, herbaceous vegetation, forest and aquatic macrophyte cover (Table 1). Lakes and secondary channels (open water) are the principal aquatic habitats that are present during low-water periods, when other floodplain areas dry out (Goulding 1980). During the annual flood pulse, areas covered by forest or herbaceous vegetation provide important food resources for many fishes, as well as spawning and nursery habitat. Herbaceous vegetation communities dominate the margins of lakes and channels; this land cover category was defined to include areas with grass, forbs, or bare soil during low-water periods. The forest category includes tall forest remnants (with emergent canopy heights to 40 m), regenerating forests (locally termed *capoeiras*) and pioneer forests, the latter two categories having lower and more variable canopy heights (Renó et al. 2011). The shrubby aroid *Montrichardia arborescens*, 1 to 4 m in height, often dominates pioneer forests in areas of low elevation and was included within the forest category.

Aquatic macrophyte cover within open-water and herbaceous areas comprised emergent or floating grasses (primarily, *Hymenachne amplexicaulis*, *Paspalum repens, P. fasciculatum, Oryza perennis,* and *Echinochloa polystachya*) or broad-leaved herbaceous plants (including *Eichhornia* spp., *Pistia* spp. *Salvinia* spp., and *Victoria* spp.). These floating meadows are highly variable seasonally and interannually, and attain their peak extent during the early rising-water period (December to mid-January; Silva et al. 2010).

Forest, open water and herbaceous vegetation at low-water stage was mapped by L. Hess (unpublished data) at 30-m resolution using two contiguous Landsat Thematic Mapper images acquired on 30 November 2008 and 23 October 2009. Landsat imagery was obtained from the

USGS Earth Explorer site (http://earthexplorer.usgs.gov/). The two contiguous Landsat Thematic Mapper images used to map floodplain land cover correspond to river stages of 2.14 m and 1.75 m at the Óbidos gauge. Aquatic macrophyte coverage (Table 1) was mapped using seven ALOS PALSAR swaths (fine-beam, HH-polarized, L-band synthetic aperture radar; resampled from 12.5 to 30 m) acquired during the early rising-water period in each of five years from 2006 to 2010. ALOS PALSAR imagery was obtained from the Alaska Satellite Facility's Vertex Data Portal (https://vertex.daac.asf.alaska.edu/).

Land-cover data obtained from remotely sensed imagery were assembled according to spatial units defined as local catchments (or *"lake systems"*). Each local catchment contains lakes, interconnecting channels, forest, and areas with herbaceous vegetation and aquatic macrophytes that are hydrologically connected for about six to nine months per year (Arantes et al. 2018). Local catchments are separated from each other by major secondary channels (areas of low elevation) and/or natural levees (areas of high elevation). We mapped 20 local catchments (Fig. 1, median area: 23.4 km^2), which encompassed a gradient of forest cover, ranging from 3 to 70%. The macrophyte metric used here was the percent of the catchment having macrophyte cover during the early-rising water period during three or more of the five years imaged (Table 1).

Field surveys

Field surveys were conducted during five expeditions covering four different stages of the annual hydrological cycle at 462 habitat areas (lakes and secondary channels (open water), and flooded herbaceous and forest) within the 20 local catchments (Fig. 1). For each habitat type within each local catchment, and during two dry periods and one rising-, high-, and falling-water period, we collected fish using a standard set of nets with different mesh sizes (11 gillnets measuring 25 x 2 meters, with mesh sizes 20, 30, 40, 50, 60, 70, 80, 90, 100, 120, and 130 mm, and one gillnet measuring 100 x 3 meters, with 180 mm mesh) to catch multiple fish size classes and species. For each season and all habitats sampled within each local catchment, average gillnet sampling effort was approximately 25 hours (SD ~4 hours). For the same seasons and habitats within each local catchment where we collected fish, we measured water temperature, dissolved oxygen concentration, depth and transparency (Table 1). We also estimated the area covered by aquatic macrophytes via visual observations (Table 1). Whereas macrophyte indices obtained via remotely sensed imagery provided large-scale estimates of coverage in local catchments, visual estimates were useful for characterizing an important feature of fish habitat at a local scale matching that of our fish assemblage surveys.

Species classification

We tested relations between forest coverage and fish biomass (CPUE) based on 25 fish groups distributed within 6 categories (Table S1). Measures of total fish biomass in local habitats involved subsets of the 186 species that were surveyed in the region. Species were grouped according to their degree of importance in local fisheries and this classification was based on their relative contribution to total yields landed in the main cities in the lower Amazon (Isaac et al. 2016, Castello et al. 2017). Fish of *high-importance* (28 species, 11 common names) contributed $\geq$ 85% of the total landing. Fish of *medium-importance* (83 species, 31 common names) contributed with 15% of the total landing. Both groups (high-importance and medium-importance) contain important species for subsistence fisheries in the Amazon (e.g., *Colossoma macropomum, Cichla monoculus, Prochilodus nigricans, Mylossoma* spp. *Myleu*s spp.) (Batista et al. 2008). Seventy-four species (39 common names) were classified as having *low-importance*

and were rarely landed for sale as food, although some of them are used as bait or sold as ornamental fish.

The other categories comprised functional groups based on trophic, migratory, life history, and swimming/microhabitat-use strategies. We classified species according to eight trophic strategies based on dietary information from published reports (Table S1). *Herbivores* (18 species) feed predominantly on C3 or C4 plant material (seeds, fruits or leaves) and on filamentous algae. *Omnivores* (47 species) ingest combinations of plant material, detritus, and invertebrates. *Detritivores* (28 species) predominantly ingest fine particulate organic matter and non-living macrophyte tissues, but also on filamentous algae. *Invertivores* (23 species) ingest variable fractions of aquatic and terrestrial insects, microcrustaceans from the benthos or water column, spiders, shrimps, and mollusks. *Planktivores* (10 species) ingest phytoplankton, zooplankton, and occasionally small amounts of plant material and detritus. *Piscivores* (45 species) ingest adult, juvenile, or larval fish, either whole or in pieces, including scales and fins. *Piscivores-macroinvertivores* (14 species) feed on the same sources as piscivores but also ingest significant fractions of diverse terrestrial or aquatic macroinvertebrates (e.g., Ephemeroptera, Chironomidae, Coleoptera, Crustacea, etc.).

We classified species according to four migratory strategies based on information on dispersal behavior from published reports. Migratory strategies of Amazon fish often are related to reproduction and/or feeding ecology and influenced by seasonal hydrology and physical-chemical conditions of habitats in the riverscape. *Sedentary* (55 species) were resident species that spend their entire life-cycles within floodplain habitats eventually performing short-distances movements. Sedentary species were small-bodied species, or had territorial behavior, or are known to be strongly associated with substrates or complex structured habitat (e.g., tree branches and aquatic vegetation). Species performing *local migration* (120) comprised a diverse group of fishes that migrate laterally from floodplain lakes or river channels onto flooded floodplain habitats following closely the dynamic 'pulsing' of water levels (Junk et al. 1989; Fernandes 1997; Carolsfeld 2003; Castello 2008). Species performing *regional migration* (8 species) migrate onto flooded floodplains habitats during high waters, but also conduct longitudinal migrations (often hundreds of kilometers) along river channels to spawn, particularly during falling waters (Goulding 1980; Ribeiro de Brito and Petrere 1990; Benedito-Cecilio and Araujo-Lima 2002; Barthem and Fabré 2004). *Long-distance* migrators (3 species) were species that migrate thousands of kilometers along river channels, though their juveniles often inhabit floodplain lakes (Barthem et al. 1991, 2017).

We classified species according to six life history strategies based on maximum body size, size at maturation, batch fecundity, and parental investment per individual offspring (Table S1) (following Winemiller & Rose (1992) and Röpke et al. (2017)). Life history strategies identify suites of intercorrelated functional traits and their associations with patterns of environmental variation involving abiotic factors, disturbance regimes, resource availability and quality, population density, risk of predation or parasitism, and challenges for dispersal (Winemiller & Rose 1992, Winemiller 2005). Sixteen species were classified as *equilibrium strategists with maturation at small size* (<120 mm standard length, SL), having low batch fecundity, large oocytes, well-developed parental care, and maximum body size between 97 - 269 mm SL. Sixteen species were *equilibrium strategists with maturation at large size* (>170 mm SL), with low batch fecundity, large oocytes, well-developed parental care and maximum size >400 mm SL. Seventy-three species were *periodic strategists with maturation at small size* (between 63 – 148 mm SL), having varied batch fecundity size (average ~ 4,000), small oocytes,

maximum size between 137 – 410 mm SL and no parental care. Forty-three species were *periodic strategists with maturation at large size* (>164 mm SL), with batch fecundity highly variable, small oocytes, no parental care and maximum size > 253 mm SL. Thirty-two species classified as *intermediate strategists* had batch fecundity between 1,000 and 9,000, relatively large oocytes, and intermediate development of parental care. Five species classified as *opportunistic* had small size (between 26–113 mm SL), early maturation (<60 mm SL), high and sustained reproductive effort but low batch fecundity and no parental care (Röpke et al. 2017).

Finally, we classified species according to five strategies of swimming/microhabitat use based on morphological traits. We based our classification on the classification of Arantes et al. (2017) that uses traits associated with swimming performance and vertical position within the water column during foraging, phenotypes that influence fitness along gradients of habitat structural complexity and other environmental features (Gatz 1979, Winemiller 1991). *Nektonic maneuverable* fishes (41 species) had laterally compressed bodies and superior mouth position, whereas *nektonic burst swimmers* (18 species) had fusiform bodies and terminal mouth position. Both groups had morphological traits associated with efficient swimming performance based on a hydrodynamic body and feeding within the water column. *Surface dwellers* (2 species) had intermediate lateral body compression, superior mouths and either deep or fusiform bodies. *Epibenthic maneuverable* fishes (57 species) were a diverse group having relatively deep bodies that are less hydrodynamic than nektonic maneuverable fishes but efficient in making lateral and vertical turns. The two last groups also had more dorsally than laterally positioned eyes. Most *Benthic-slow* (36 species) and *Benthic-fast* (23 species) had relatively wide bodies, dorsally located eyes, and inferior mouths, which are characteristic of bottom dwellers. Benthic-fast fishes had higher muscle mass and larger pectoral and caudal fins ratio areas than benthic-slow fishes; morphological traits associated with increasing swimming performance. A few benthic-fast (1 species) and benthic-slow (3 species—e.g., *Hoplias malabaricus*) fishes had terminal or superior mouths. *Gymnotiformes* (8 species) comprised a diverse group of electric fishes, either substrate or aquatic vegetation dwellers, that are inactive during daylight but actively forage during the night using weak electric organ to locate their prey (Fernandes et al. 2004; Carvalho et al. 2009). As mentioned herein, we did not include gymnotiforms, long-distance migrators and opportunistic strategists in the analyses due to their small sample size and complete absence at some levels of the categorical variables (i.e., seasons and habitat types).

Statistical Analyses

Modelling associations of fish biomass and forest

Fish biomass (CPUE) for each fish group was modeled as a function of linear predictors within a generalized linear model (GLM) framework using a Poisson-Gamma distribution:

$$g(y_i) = \alpha + \boldsymbol{l_i}\boldsymbol{\varphi} + \boldsymbol{e_i}\boldsymbol{\beta} + \delta s_i + \theta h_i + \gamma m_i + \boldsymbol{\varepsilon_i}$$

where, g() is the log link function, *i* represents the data sampling habitat, y is fish biomass standardized by sampling effort (CPUE), $\boldsymbol{l}$ is a row vector of three measures of land cover (forest, open water, large-scale estimate of aquatic macrophyte cover), $\boldsymbol{e}$ is a row vector of two first PCA axis representing environmental covariates (PC1 represent gradients of transparency and dissolved oxygen and PC2 macrophyte cover, temperature and depth, Fig S1), s is a season factor variable, h is the habitat type factor, m is an indicator for the presence of management, and ε is the error term. Frequent zero catches, such as observed for our CPUE data, is a common issue in fishery modeling that have been addressed in a straightforward manner within the GLM framework by using a Poisson-Gamma distribution from the family Tweedie, the set of exponential distributions indexed by a power parameter (Jorgensen 1987; Peel et al. 2013). This distribution handles zero values uniformly with positive and continuous values and it was found to outperform other models used for CPUE data containing a point mass at zeros (e.g., delta models, generalized linear models with an additive constant) (Shono 2008; Carvalho et al. 2010; Li et al. 2011). For a random variable Y that is distributed Tweedie, E(Y) = μ and Var(Y) = $\varphi\mu^p$ where μ is the mean of the distribution, φ is the dispersion parameter, and *p* is an extra parameter (power parameter) that controls the variance of the distribution. The Tweedy family of distribution include the Normal (when p=0), Poisson (p=1) and Gamma (p=2) distributions. When, $p \in (1,2)$, such as in our study, the Tweedie distribution assumes the form of a compound Poisson-Gamma distribution, which allows modelling a variable that has both discrete and continuous components.

We assessed the quality of models fit via visual inspection of plots of model residuals. We used randomized quantile residuals as recommended by Dunn and Smyth (1996) and Dunn (2009) for model fits using the Tweedie family, and as used in several cases of fishery modeling studies (e.g.,Tascheri et al. 2010; Peel et al. 2013). The randomized quantile residuals were examined for heteroscedasticity and approximate normality. Because the data have a non-Normal nature, Pearson and deviance residuals are intrinsically non-Normal, and difficult to interpret due to a large proportion of exact zeros (Peel et al. 2013). Instead, quantile residuals have an exact Normal distribution provided if correct response distribution is used and indications of non-Normality are interpretable as deficiencies in the model (Dunn 2009).

Spatial dependence is a common feature of ecological studies because data collected at sites that are located closer to each other tend to be more similar than data collected from sites that are farther apart (Legendre and Fortin 1989). Therefore, we also tested for spatial autocorrelation of the models' residuals using Moran's I statistics. Moran's I is a correlation coefficient that measures the overall spatial autocorrelation of the data (I=-1 indicates a perfect dispersion, I=0 indicates a perfect randomness, and I=1 a perfect clustering) (Sokal and Oden 1978). We tested whether Moran's statistics values differ from random by comparing the observed Moran's I from each model residuals with bootstrapped generated Moran's I. The Moran's I statistic was bootstrapped by randomly assigning longitude and latitude values to the

residuals values and Kernel density estimates of the Moran's I statistics were used to calculate a 95% highest density region (i.e., confidence interval). Moran's I results indicated that data is not strongly dependent upon space across distances (see Figures S2); therefore, incorporating spatial autocorrelation was not a concern for our models.

Analyses were performed in R v. 3.3.3. Models were fitted using the *statmod* (Giner and Smyth 2016) and *Tweedie* (Dunn and Smyth 2005) packages and Moran's I were calculated using the *ape* (Paradis et al. 2004), *geoR* (Ribeiro Jr et al. 2001) and *fields* (Nychka et al. 2005) packages.

Supplementary references

Arantes, C.C., Castello, L., Cetra, M. and Schilling, A. (2013) Environmental influences on the distribution of arapaima in Amazon floodplains. *Environmental Biology of Fishes* **96**, 1257–1267.

Barbarino, D. and Winemiller, K. (2003) Dietary segregation among large catfishes of the Apure and Arauca Rivers, Venezuela. *Journal of Fish Biology* **63**, 410–427.

Barthem, R.B., de Brito Ribeiro, M.C.L. and Petrere, M. (1991) Life strategies of some long-distance migratory catfish in relation to hydroelectric dams in the Amazon Basin. *Biological conservation* **55**, 339–345.

Barthem, R.B. and Fabré, N.N. (2004) Biologia e diversidade dos recursos pesqueiros da Amazônia. *A pesca e os recursos pesqueiros na Amazônia brasileira* **1**, 17–62.

Barthem, R.B., Goulding, M., Leite, R.G., et al. (2017) Goliath catfish spawning in the far western Amazon confirmed by the distribution of mature adults, drifting larvae and migrating juveniles. *Scientific Reports* **7**.

Benedito-Cecilio, E. and Araujo-Lima, C. (2002) Variation in the carbon isotope composition of Semaprochilodus insignis, a detritivorous fish associated with oligotrophic and eutrophic Amazonian rivers. *Journal of Fish Biology* **60**, 1603–1607.

Carolsfeld, J. (2003) *Migratory fishes of South America: biology, fisheries and conservation status*. Idrc.

Carvalho, F.C., Murie, D.J., Hazin, F.H., Hazin, H.G., Leite-Mourato, B., Travassos, P. and Burgess, G.H. (2010) Catch rates and size composition of blue sharks (*Prionace glauca)* caught by the Brazilian pelagic longline fleet in the southwestern Atlantic Ocean. *Aquatic Living Resources* **23**, 373–385.

Carvalho, L.N., Zuanon, J. and Sazima, I. (2009) . *Tropical Biology and Conservation Management. Oxford: Case studies, Eolss Publishers Co. Ltd*, 113–144.

Castello, L. (2008) Lateral migration of Arapaima gigas in floodplains of the Amazon. *Ecology of Freshwater Fish* **17**, 38–46.

Correa, S.B. and Winemiller, K.O. (2014) Niche partitioning among frugivorous fishes in response to fluctuating resources in the Amazonian floodplain forest. *Ecology* **95**, 210–224.

Dunn, P.K. (2009) Improving comparisons between models for CPUE. *Fisheries Research* **97**, 148–149.

Dunn, P.K. and Smyth, G.K. (1996) Randomized quantile residuals. *Journal of Computational and Graphical Statistics* **5**, 236–244.

Dunn, P.K. and Smyth, G.K. (2005) Series evaluation of Tweedie exponential dispersion model densities. *Statistics and Computing* **15**, 267–280.

Fernandes, C. (1997) Lateral migration of fishes in Amazon floodplains. *Ecology of freshwater fish* **6**, 36–44.

Fernandes, C.C., Podos, J. and Lundberg, J.G. (2004) Amazonian ecology: tributaries enhance the diversity of electric fishes. *Science* **305**, 1960–1962.
Giner, G. and Smyth, G.K. (2016) statmod: Probability Calculations for the Inverse Gaussian Distribution. *arXiv preprint arXiv:1603.06687*.
Goulding, M. (1980) *The fishes and the forest: explorations in Amazonian natural history*. Univ of California Press.
Jorgensen, B. (1987) Exponential dispersion models. *Journal of the Royal Statistical Society. Series B (Methodological)*, 127–162.
Junk, W.J., Bayley, P.B., Sparks, R.E. and others (1989) The flood pulse concept in river-floodplain systems. *Canadian special publication of fisheries and aquatic sciences* **106**, 110–127.
Legendre, P. and Fortin, M.J. (1989) Spatial pattern and ecological analysis. *Vegetatio* **80**, 107–138.
Li, Y., Jiao, Y. and He, Q. (2011) Decreasing uncertainty in catch rate analyses using Delta-AdaBoost: An alternative approach in catch and bycatch analyses with high percentage of zeros. *Fisheries research* **107**, 261–271.
Lopes, C., Benedito, E. and Martinelli, L. (2009) Trophic position of bottom-feeding fish in the Upper Paraná River floodplain. *Brazilian Journal of Biology* **9**, 573–581.
Mérona, B. de and Mérona, J. (2004) Food resource partitioning in a fish community of the central Amazon floodplain. *Neotropical Ichthyology* **2**, 75–84.
Nychka, D., Furrer, R. and Sain, S. (2005) fields: Tools for spatial data. *National Center for Atmospheric Research*.
Paradis, E., Claude, J. and Strimmer, K. (2004) APE: analyses of phylogenetics and evolution in R language. *Bioinformatics* **20**, 289–290.
Peel, D., Bravington, M., Kelly, N., Wood, S.N. and Knuckey, I. (2013) A model-based approach to designing a fishery-independent survey. *Journal of Agricultural, Biological, and Environmental Statistics*, 1–21.
R Core, T. (2017) R: A language and environment for statistical computing. R Foundation for Statistical Computing, Vienna, Austria. *Online: http://www. R-project. org*.
Ribeiro de Brito, M.C.L. and Petrere, M. (1990) Fisheries ecololgy and management of the Jaraqui (Semaprochilodus Taeniurus, S. Insignis) in central Amazonia. *Regulated Rivers: Research \& Management* **5**, 195–215.
Ribeiro Jr, P.J., Diggle, P.J. and others (2001) geoR: a package for geostatistical analysis. *R news* **1**, 14–18.
Röpke, C.P., Amadio, S., Zuanon, J., Ferreira, E.J., de Deus, C.P., Pires, T.H. and Winemiller, K.O. (2017) Simultaneous abrupt shifts in hydrology and fish assemblage structure in a floodplain lake in the central Amazon. *Scientific Reports* **7**, 40170.
Röpke, C.P., Ferreira, E. and Zuanon, J. (2014) Seasonal changes in the use of feeding resources by fish in stands of aquatic macrophytes in an Amazonian floodplain, Brazil. *Environmental biology of fishes* **97**, 401–414.
Santos, R., Ferreira, E. and Amadio, S. (2008) Effect of seasonality and trophic group on energy acquisition in Amazonian fish specie. *Ecology of Freshwater Fish* **17**, 340–348.
Shibuya, A. and Zuanon, J. (2013) Catfishes as prey items of Potamotrygonid stingrays in the Solimões and Negro rivers, Brazilian Amazon. *Biota Neotropica* **13**, 376–379.
Shono, H. (2008) Application of the Tweedie distribution to zero-catch data in CPUE analysis. *Fisheries Research* **93**, 154–162.

Sokal, R.R. and Oden, N.L. (1978) Spatial autocorrelation in biology: 1. Methodology. *Biological journal of the Linnean Society* **10**, 199–228.

Tascheri, R., Saavedra-Nievas, J. and Roa-Ureta, R. (2010) Statistical models to standardize catch rates in the multi-species trawl fishery for Patagonian grenadier (Macruronus magellanicus) off Southern Chile. *Fisheries Research* **105**, 200–214.

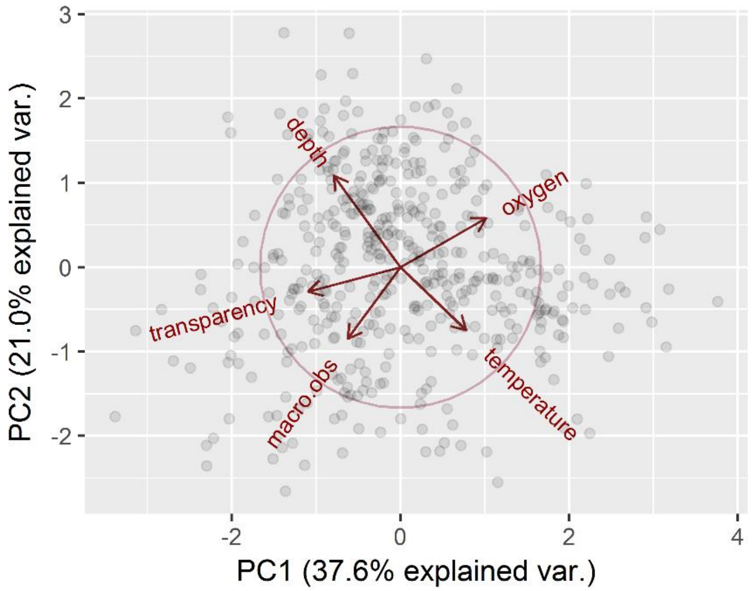

Figure S1. Principal components analysis with habitats ordination according to the local environmental variables: depth, dissolved oxygen, transparency, temperature and the local-scale estimate of aquatic macrophyte cover (macro.obs) (Table 1). PC1 is associated with a gradient of transparency (score -2.4) --dissolved oxygen (score 2.2) and PC2 with a gradient of macrophyte (score -1.6) and temperature (score -1.4) --depth (score 2.0). Data were standardized and PCA was performed using *stats* library and *prcomp* function in R software (R Core team 2017)

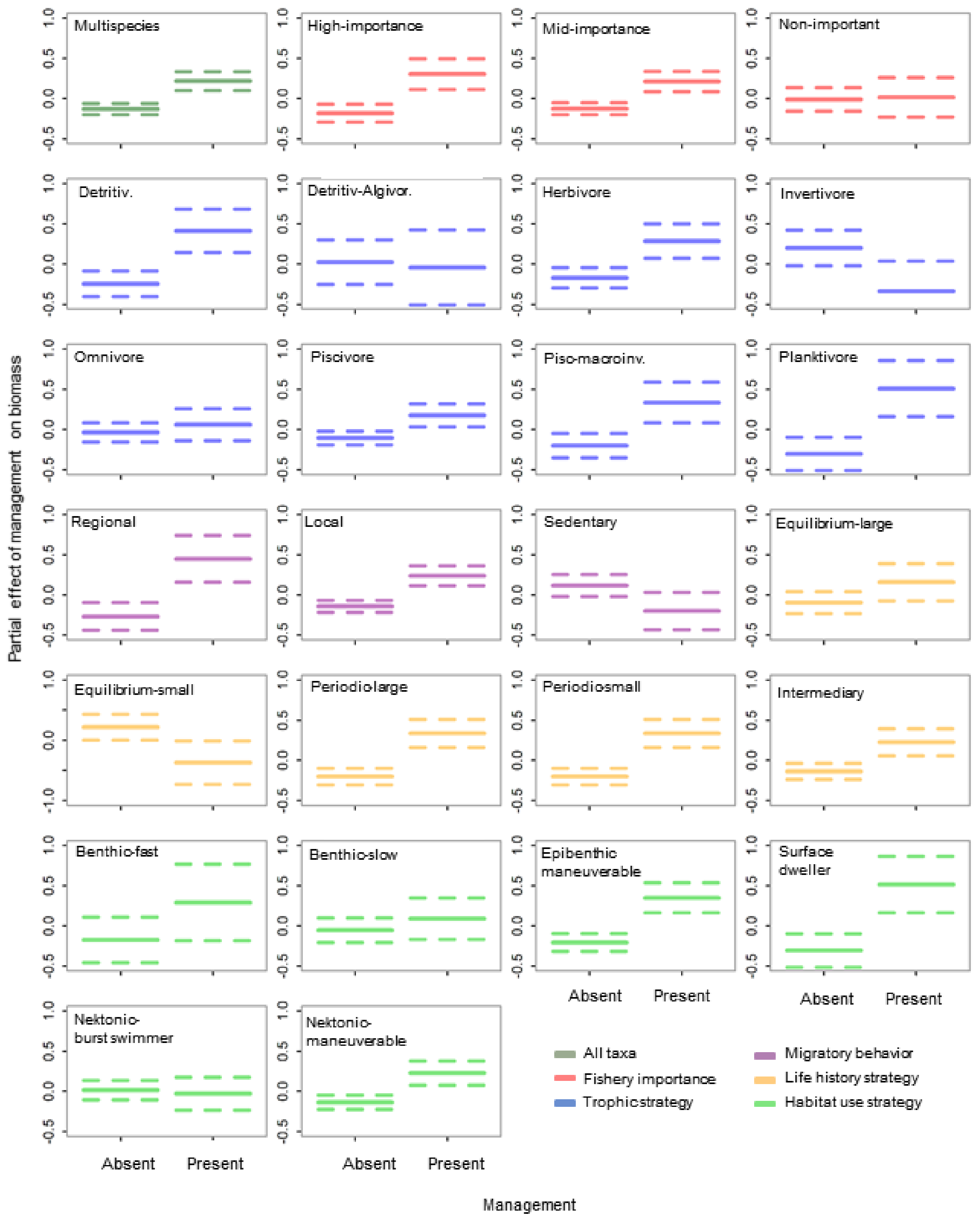

Figure S2. Partial effects of management on relative biomass (CPUE) for each fish group. The reference level is absence of management (coefficient = 0), meaning that the coefficient size for presence of management reflect its size being compared to that of absence of management while controlling for the effects of other variables.

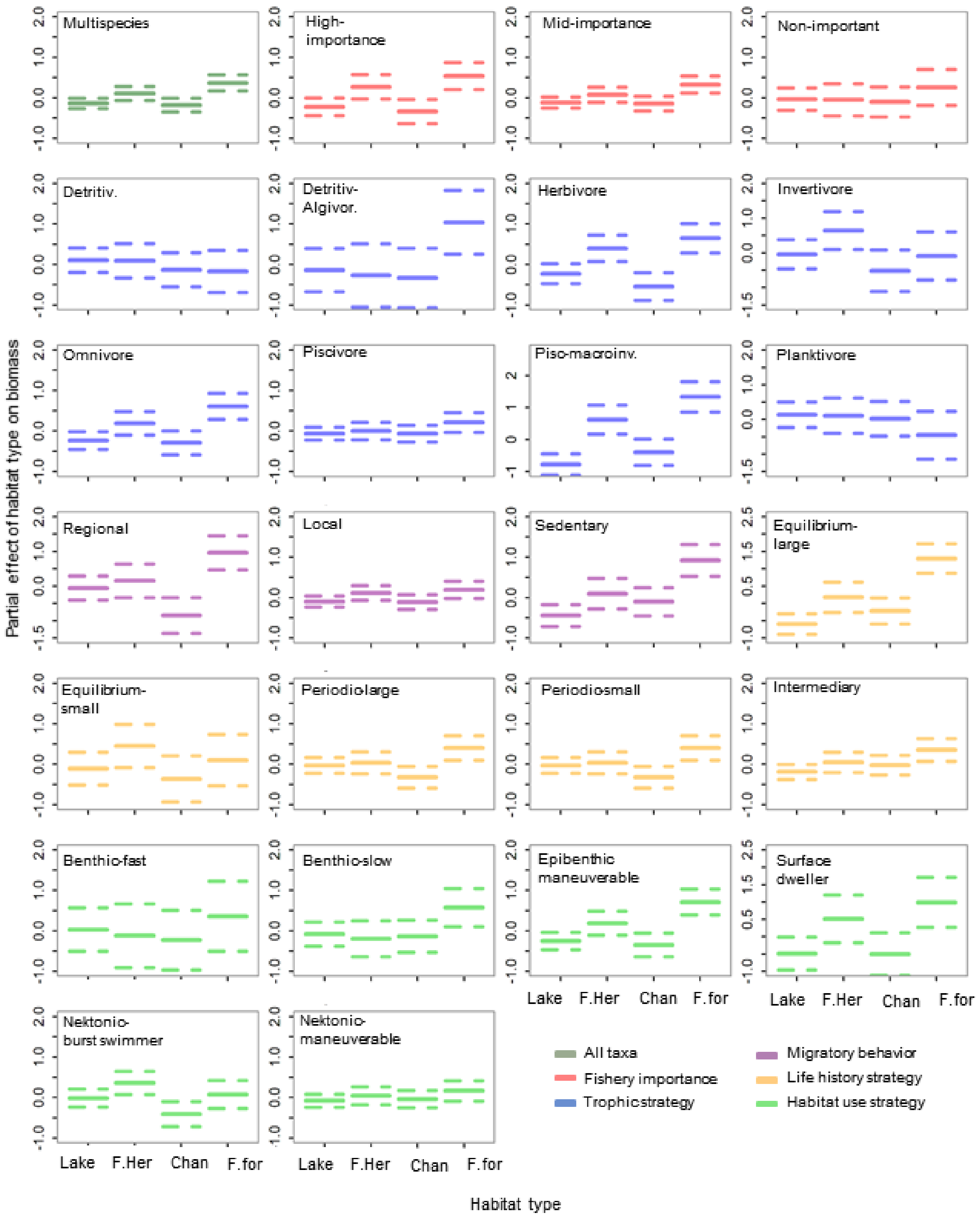

Figure S3. Partial effects of habitat type on relative biomass (CPUE) for each fish group. Habitat type are: Lake, Flooded herbaceous (F.her), Channel (Chan), and Flooded forest (F.for) (See Table 1). The reference level is lake habitat type (coefficient = 0), meaning that the coefficient size for the other habitat types reflect their size being compared to that of lake while controlling for the effects of other variables.

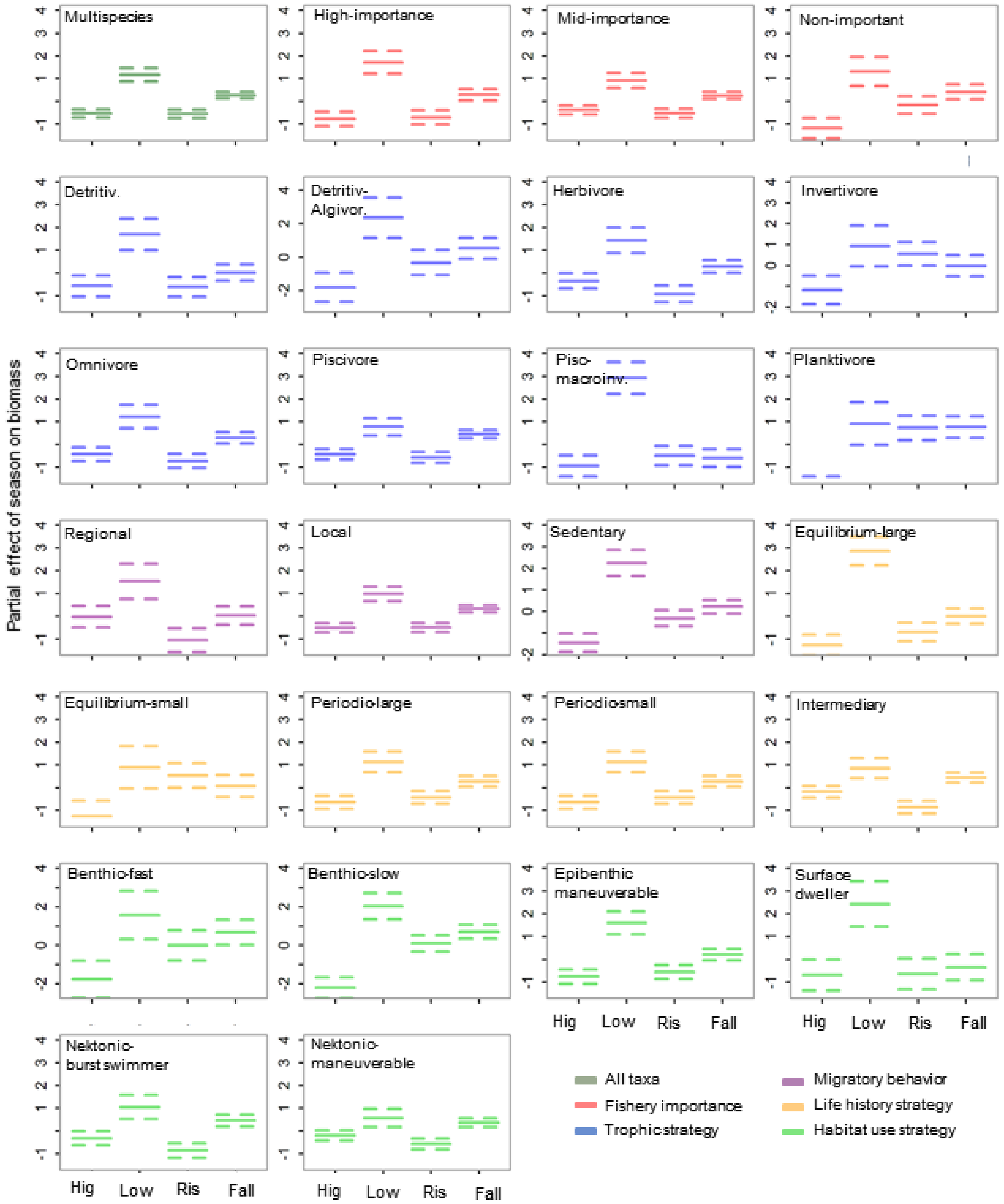

Figure S4. Partial effects of season on relative biomass (CPUE) for each fish group. Season are: High water (Hig), Low water (Low), Rising water (Ris), and Falling water (Fall) (See Table 1). The reference level is high water (coefficient = 0), meaning that the coefficient size for the effect other seasons reflect their size being compared to that of high water season while controlling for the effects of other variables.

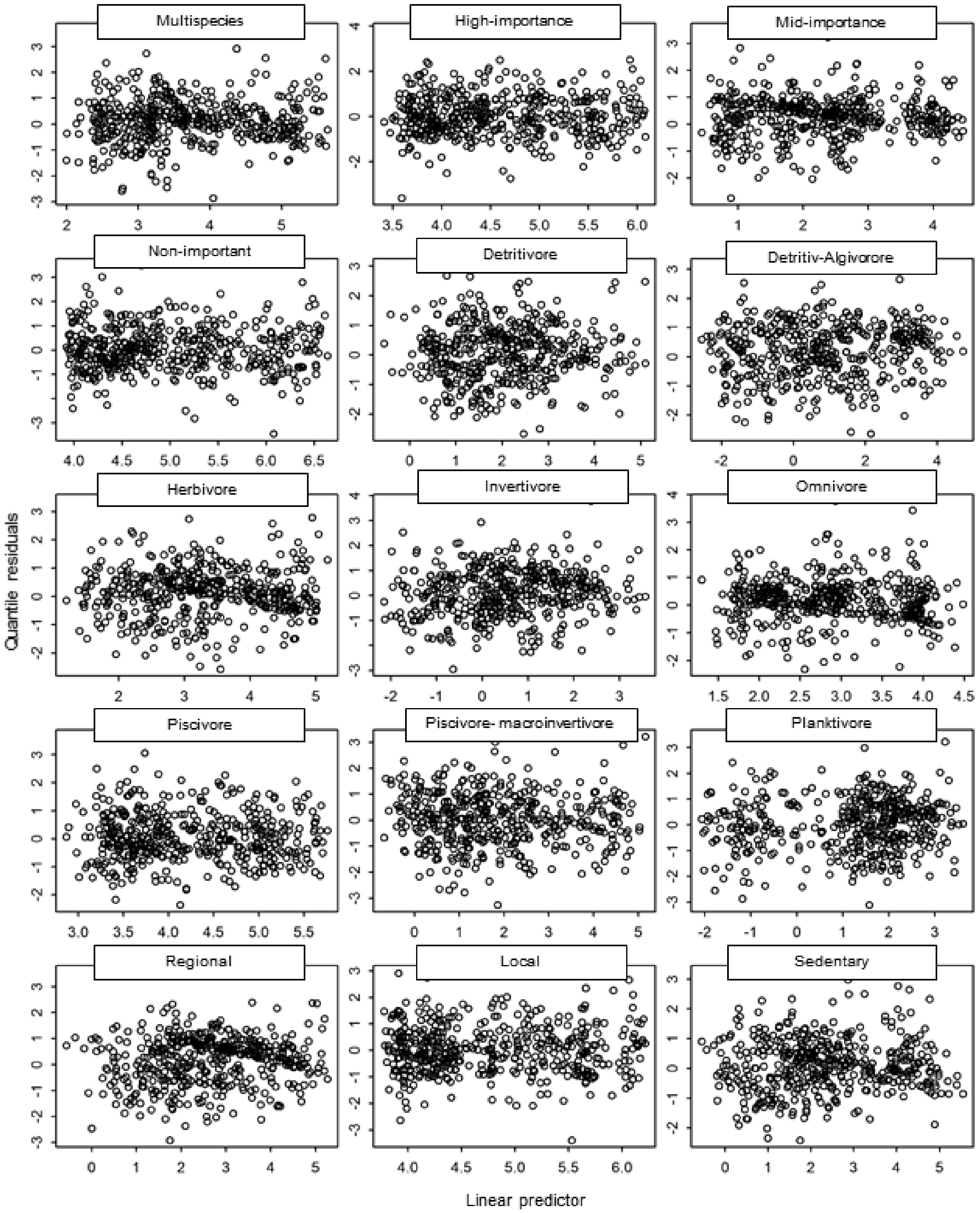
Multispecies
High-importance
Mid-importance
Non-important
Detritivore
Detritiv-Algivorore
Herbivore
Invertivore
Omnivore
Piscivore
Piscivore- macroinvertivore
Planktivore
Regional
Local
Sedentary
Quantile residuals
Linear predictor

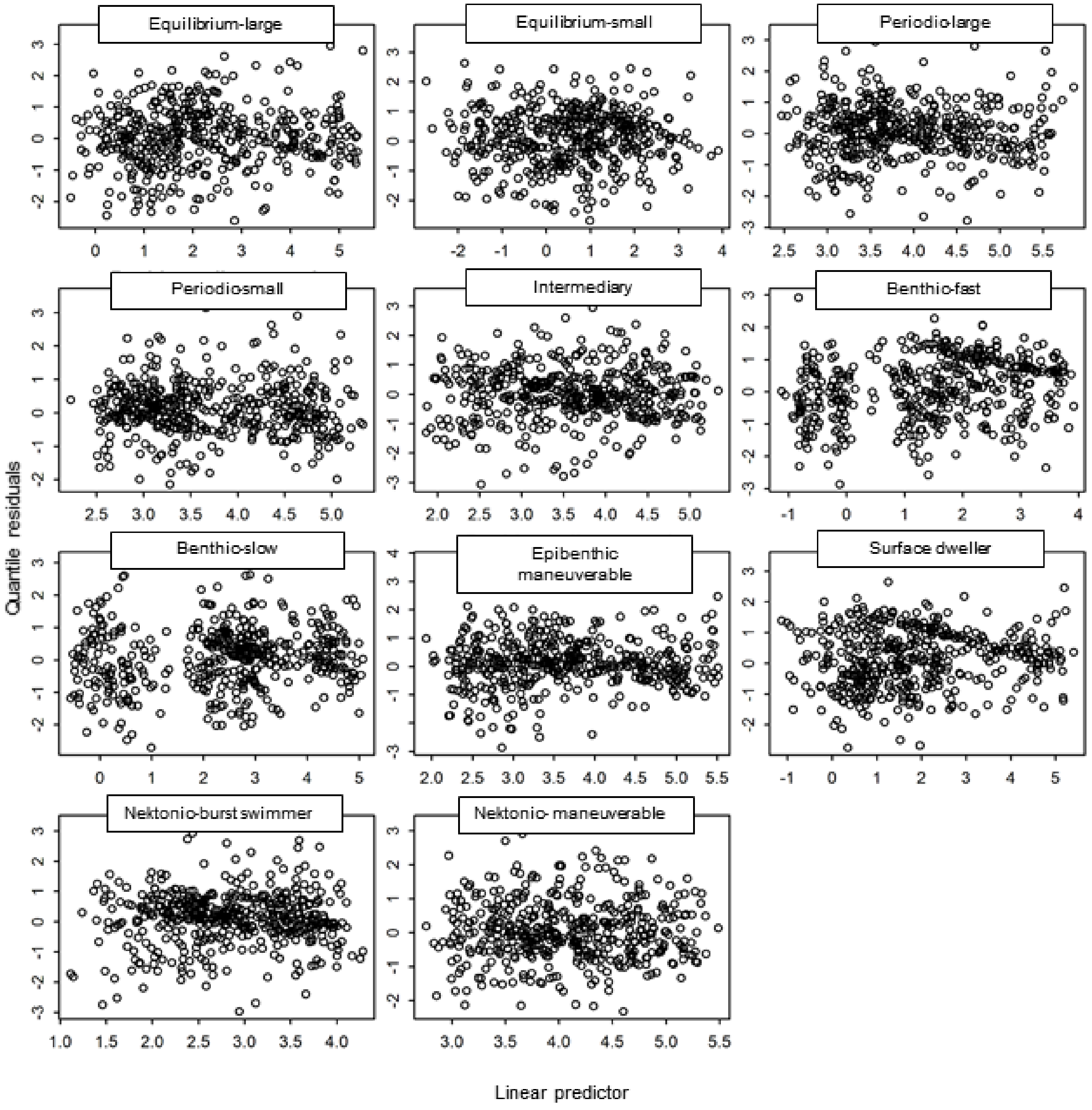

Figure S5. Randomized quantile residuals versus linear predictor for all models. Fish category is indicated on the top of the graphs (see Table S1).

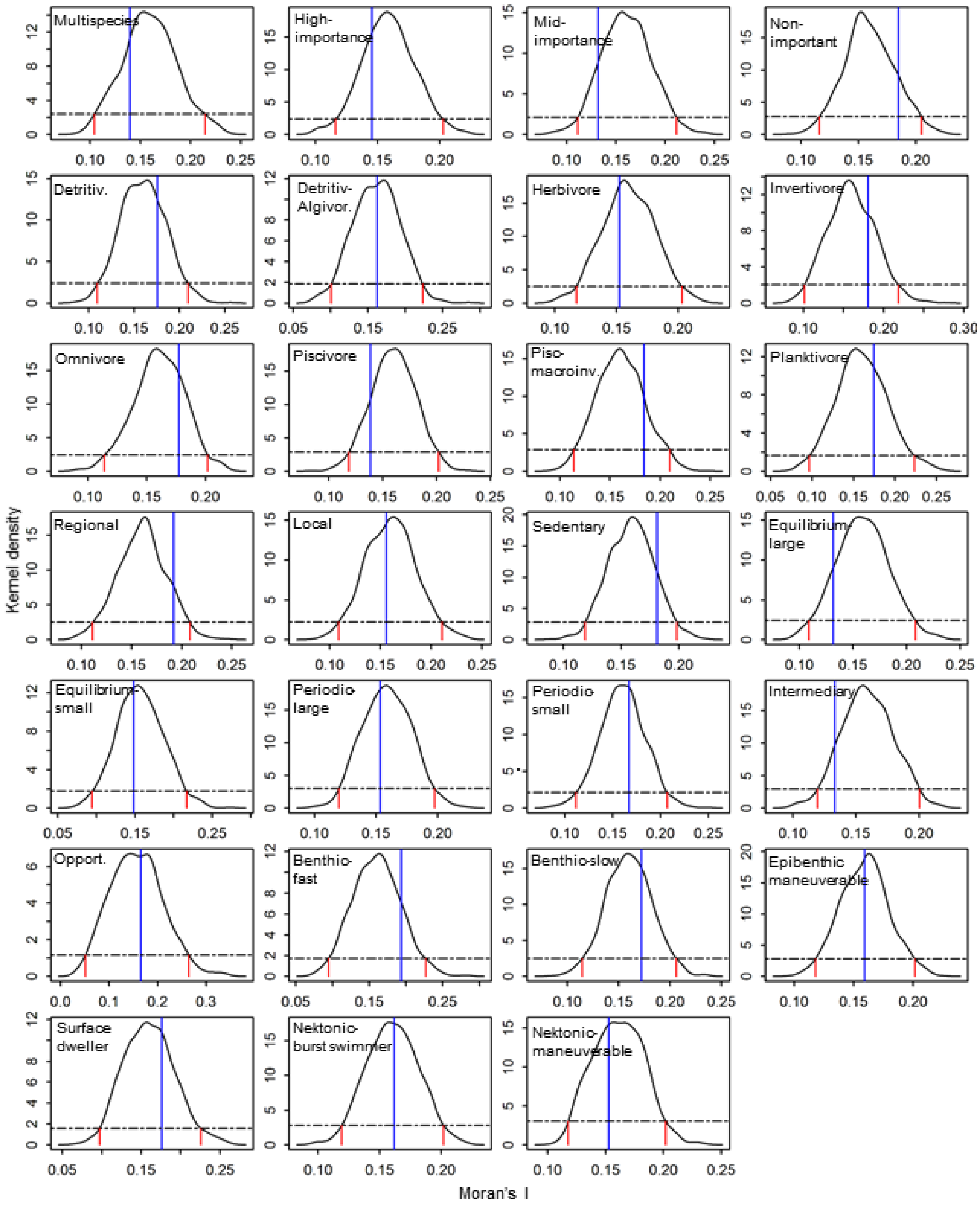

Figure S6. Observed values of Moran' I (blue line) and kernel density estimate (essentially a smoothed histogram) of bootstrapped Moran's I generated by randomly assigning the latitude and longitude values to residuals values. The red vertical lines show the limits of the 95% confidence interval for the bootstrapped Moran's I. Observed Moran's I values were generally within confidence intervals indicating a lack of spatial dependence.

Table S1- Fish species (common and scientific names and families) and their degree of importance for regional fisheries, and trophic, migratory, life history and microhabitat use strategies. Trophic strategies classification was based on Barbarino and Winemiller (2003) Mérona and Mérona (2004), Santos et al. (2008), Shibuya and Zuanon (2013), Röpke et al. (2014), Correa and Winemiller (2014), Lopes et al. (2009) and Röpke et al. (2017). Migratory behavior classification was based on Goulding (1980), Junk et al. (1989), Ribeiro de Brito and Petrere (1990), Barthem et al. (1991), Fernandes (1997), Benedito-Cecilio and Araujo-Lima (2002), Carolsfeld (2003), Barthem and Fabré (2004), Castello (2008), Arantes et al. (2013) and Barthem et al. (2017). Life history classification was based on Röpke et al. (2017), and microhabitat use classification was based on Arantes et al. 2017 (see cited literature and supplementary references and Material and Methods). Piscivore/Macroinvertivore = Pisc./MacInv; Equilibrium with maturation at large size = Equilibrium-large; Equilibrium with maturation at small size = Equilibrium-small; Periodic with maturation in large size = Periodic-large; Periodic with maturation at small size=Periodic-small; Epibenthic maneuverable =Epib. maneuv.; Nektonic maneuverable=Nekt maneuv; Nektonic burst swimmers=Nekt swim; Surface

Common name	Family<br>Species	Fishery importance	Trophic	Migratory	Life history	Habitat use
	Anostomidae					
Aracu	*Laemolyta cf. proxima*	High	Herbivore	Local	Periodic-small	Nekt swim
	Laemolyta taeniata	High	Herbivore	Local	Periodic-small	Nekt swim
	Leporinus bimaculatus	High	Omnivore	Local	Periodic-small	Epib maneuv
	Leporinus fasciatus	High	Omnivore	Local	Periodic-large	Epib maneuv
	Leporinus friderici	High	Omnivore	Local	Periodic-large	Epib maneuv
	Leporinus trifasciatus	High	Omnivore	Local	Periodic-large	Epib maneuv
	Leporinus wolfei	High	Omnivore	Local	Periodic-large	Epib maneuv
	Pseudanos gracilis	High	Omnivore	Local	Periodic-small	Epib maneuv
	Rhytiodus argenteofuscus	High	Herbivore	Local	Periodic-small	Nekt swim
	Rhytiodus microlepis	High	Herbivore	Local	Periodic-small	Nekt swim
	Schizodon fasciatus	High	Herbivore	Local	Periodic-small	Epib maneuv
	Schizodon vittatus	High	Herbivore	Local	Periodic-small	Epib maneuv
	Loricariidae					
Acari/bodo	*Pterygoplichthys gibbiceps*	High	Detrit./alg.	Sedentary	Equilibrium-large	Benthic-slow
	Pterygoplichthys pardalis	High	Detritivore	Sedentary	Equilibrium-large	Benthic-slow
	Pimelodidae					
Piracatinga	*Calophysus macropterus*	High	Piscivore	Local	Periodic-large	Benthic-fast
Mapara	*Hypophthalmus edentatus*	High	Planktivore	Local	Periodic-large	Epib maneuv
	Hypophthalmus fimbriatus	High	Planktivore	Local	Periodic-large	Epib maneuv
	Hypophthalmus marginatus	High	Planktivore	Local	Periodic-large	Epib maneuv

Table S1: Continued

Common name	Family<br>Species	Fishery importance	Trophic	Migratory	Life history	Habitat use
Fura calça	*Pimelodina flavipinnis*	High	Piscivore	Local	Periodic-large	Benthic-fast
Surubim	*Pseudoplatystoma fasciatum*	High	Piscivore	Local	Periodic-large	Benthic-fast
Caparari/ Surubim	*Pseudoplatystoma fm*	High	Piscivore	Local	Periodic-large	Benthic-fast
	Prochilodontidae					
Curimata	*Prochilodus nigricans*	High	Detritivore	Regional	Periodic-large	Epib maneuv
Jaraqui	*Semaprochilodus insignis*	High	Detritivore	Regional	Periodic-large	Epib maneuv
	Semaprochilodus taeniurus	High	Detritivore	Regional	Periodic-large	Epib maneuv
	Sciaenidae					
Pescada	*Plagioscion auratus*	High	Piscivore	Local	Periodic-large	Epib maneuv
	Plagioscion cf. surinamensis	High	Piscivore	Local	Periodic-large	Epib maneuv
	Plagioscion squamosissimus	High	Piscivore	Local	Periodic-large	Epib maneuv
	Pachypops trifilis	High	Pisc./MacInv	Local	Periodic-large	Epib maneuv
	Serrasalmidae					
Tambaqui	*Colossoma macropomum*	High	Herbivore	Regional	Periodic-large	Epib maneuv
	Arapamidae					
Pirarucu	*Arapaima sp.*	Mid	Piscivore	Local	Equilibrium-large	Benthic-fast
	Auchenipteridae					
Mandubé	*Ageneiosus inermis*	Mid	Piscivore	Local	Periodic-large	Benthic-slow
	Auchenipterus ambyiacus	Mid	Invertivore	Sedentary	Intermediate	Epib maneuv
	Auchenipterus nuchalis	Mid	Invertivore	Sedentary	Intermediate	Epib maneuv
	Callichthyidae					
Tamoata	*Hoplosternum littorale*	Mid	Invertivore	Sedentary	Intermediate	Benthic-slow
	Characidae					
Matrinchã	*Brycon cephalus*	Mid	Omnivore	Regional	Periodic-large	Epib maneuv
	Brycon falcatus	Mid	Omnivore	Regional	Periodic-large	Epib maneuv
	Brycon melanopterus	Mid	Omnivore	Regional	Periodic-large	Epib maneuv
Sardinha	*Triportheus albus*	Mid	Omnivore	Local	Periodic-small	Nekt maneuv
	Triportheus angulatus	Mid	Omnivore	Local	Periodic-small	Nekt maneuv
	Triportheus auritus	Mid	Omnivore	Local	Periodic-small	Nekt swim

Table S1: Continued

Common name	Family Species	Fishery importance	Trophic	Migratory	Life history	Habitat use
	Cichlidae					
Acará	*Astronotus crassipinnis*	Mid	Pisc./MacInv	Sedentary	Equilibrium-large	Epib maneuv
	Astronotus ocellatus	Mid	Pisc./MacInv	Sedentary	Equilibrium-large	Epib maneuv
	Chaetobranchopsis orbicularis	Mid	Planktivore	Sedentary	Equilibrium-small	Epib maneuv
	Chaetobranchus flavescens	Mid	Planktivore	Sedentary	Equilibrium-small	Epib maneuv
	Geophagus proximus	Mid	Invertivore	Sedentary	Equilibrium-small	Epib maneuv
	Heros efasciatus	Mid	Omnivore	Sedentary	Equilibrium-small	Epib maneuv
	Mesonauta festivus	Mid	Omnivore	Sedentary	Equilibrium-small	Epib maneuv
	Mesonauta insignis	Mid	Omnivore	Sedentary	Equilibrium-small	Epib maneuv
	Satanoperca jurupari	Mid	Invertivore	Sedentary	Equilibrium-small	Epib maneuv
Tucunare	*Cichla monoculus*	Mid	Piscivore	Sedentary	Equilibrium-large	Epib maneuv
Jacunda	*Crenicichla johanna*	Mid	Pisc./MacInv	Sedentary	Equilibrium-small	Epib maneuv
	Crenicichla reticulata	Mid	Pisc./MacInv	Sedentary	Equilibrium-large	Epib maneuv
	Curimatidae					
Branquinha	*Curimata incompta*	Mid	Detritivore	Local	Periodic-small	Epib maneuv
	Curimata inornata	Mid	Detritivore	Local	Periodic-small	Epib maneuv
	Curimata vittata	Mid	Detritivore	Local	Periodic-small	Epib maneuv
	Curimatella alburna	Mid	Detritivore	Local	Periodic-small	Epib maneuv
	Curimatella meyeri	Mid	Detritivore	Local	Periodic-small	Epib maneuv
	Potamorhina altamazonica	Mid	Detritivore	Local	Periodic-small	Nekt maneuv
	Potamorhina latior	Mid	Detritivore	Local	Periodic-small	Nekt maneuv
	Potamorhina pristigaster	Mid	Detritivore	Local	Periodic-small	Nekt maneuv
	Psectrogaster amazonica	Mid	Detritivore	Local	Periodic-small	Nekt maneuv
	Psectrogaster essequibensis	Mid	Detritivore	Local	Periodic-small	Nekt maneuv
	Psectrogaster hy	Mid	Detritivore	Local	Periodic-small	Nekt maneuv
	Psectrogaster rutiloides	Mid	Detritivore	Local	Periodic-small	Nekt maneuv
	Cynodontidae					
Peixe-cachorro	*Cynodon gibbus*	Mid	Piscivore	Local	Periodic-large	Nekt maneuv
	Hydrolycus scomberoides	Mid	Piscivore	Local	Periodic-large	Nekt maneuv
	Rhaphiodon vulpinus	Mid	Piscivore	Local	Periodic-large	Nekt maneuv

Table S1: Continued

Common name	Family Species	Fishery importance	Trophic	Migratory	Life history	Habitat use
	Doradidae					
Bacu-pedra	*Lithodoras dorsalis*	Mid	Herbivore	Long-dist	Periodic-large	Benthic-fast
Rebeca	*Megalodoras uranoscopus*	Mid	Herbivore	Long-dist	Periodic-large	Benthic-fast
Cujuba	*Oxydoras niger*	Mid	Omnivore	Local	Periodic-large	Benthic-fast
Bacu	*Pterodoras granulosus*	Mid	Herbivore	Local	Periodic-large	Benthic-fast
	Erythrinidae					
Jeju	*Hoplerythrinus unitaeniatus*	Mid	Piscivore	Sedentary	Intermediate	Benthic-slow
Traira	*Hoplias malabaricus*	Mid	Piscivore	Sedentary	Intermediate	Benthic-slow
	Hemiodontidae					
Charuto/ Cubiu/Erana	*Anodus elongatus*	Mid	Planktivore	Local	Periodic-small	Nekt swim
	Hemiodus argenteus	Mid	Omnivore	Local	Periodic-small	Nekt swim
	Hemiodus goeldii	Mid	Omnivore	Local	Periodic-small	Nekt swim
Charuto	*Hemiodus immaculatus*	Mid	Omnivore	Local	Periodic-small	Nekt swim
	Hemiodus microlepis	Mid	Omnivore	Local	Periodic-small	Nekt swim
	Hemiodus unimaculatus	Mid	Omnivore	Local	Periodic-small	Nekt swim
	Heptapteridae					
Mandi	*Pimelodella cristata*	Mid	Omnivore	Local	Periodic-small	Benthic-fast
	Pimelodella geryi	Mid	Omnivore	Local	Periodic-small	Benthic-fast
	Osteoglossidae					
Aruana	*Osteoglossum bicirrhosum*	Mid	Pisc./MacInv	Local	Equilibrium-large	Surf dweller
	Pimelodidae					
Filhote	*Brachyplatystoma filamentosum*	Mid	Piscivore	Long-dist	Periodic-large	Benthic-fast
Pirarara	*Phractocephalus hemioliopteracus*	Mid	Piscivore	Local	Periodic-large	Benthic-fast
Mandi	*Pimelodus blochii*	Mid	Omnivore	Local	Periodic-small	Benthic-fast
Piranambu	*Pinirampus pirinampu*	Mid	Piscivore	Local	Periodic-large	Benthic-fast
Cara de gato	*Platynematichthys notatus*	Mid	Piscivore	Local	Periodic-large	Benthic-fast

Table S1: Continued

Common name	Family<br>Species	Fishery importance	Trophic	Migratory	Life history	Habitat use
	Potamotrygonidae					
Arraia	*Potamotrygon motoro*	Mid	Pisc./MacInv	Local	Equilibrium-large	Benthic-fast
	Potamotrygon sp.	Mid	Pisc./MacInv	Local	Equilibrium-large	Benthic-fast
	Pristigasteridae					
Apapa	*Ilisha amazonica*	Mid	Planktivore	Local	Periodic-small	Nekt maneuv
	Pellona castelnaeana	Mid	Piscivore	Local	Periodic-large	Nekt maneuv
	Pellona flavipinnis	Mid	Piscivore	Local	Periodic-large	Nekt maneuv
	Serrasalmidae					
Pacu	*Metynnis argenteus*	Mid	Herbivore	Local	Intermediate	Nekt maneuv
	Metynnis hypsauchen	Mid	Herbivore	Local	Intermediate	Nekt maneuv
	Metynnis luna	Mid	Herbivore	Local	Intermediate	Nekt maneuv
	Myleus rubripinnis	Mid	Herbivore	Local	Intermediate	Nekt maneuv
	Myloplus torquatus	Mid	Herbivore	Local	Intermediate	Nekt maneuv
	Mylossoma aureum	Mid	Herbivore	Local	Periodic-small	Nekt maneuv
	Mylossoma duriventre	Mid	Herbivore	Local	Periodic-small	Nekt maneuv
	Serrasalmidae					
Pirapitinga	*Piaractus brachypomus*	Mid	Herbivore	Regional	Periodic-large	Surf dweller
Piranha	*Catoprion mento*	Mid	Piscivore	Local	Intermediate	Epib maneuv
	Pristobrycon sp.	Mid	Omnivore	Local	Intermediate	Nekt maneuv
	Pristobrycon striolatus	Mid	Omnivore	Local	Intermediate	Nekt maneuv
	Pygocentrus nattereri	Mid	Piscivore	Local	Intermediate	Nekt maneuv
	Serrasalmus eigenmanni	Mid	Piscivore	Local	Intermediate	Nekt maneuv
	Serrasalmus elongatus	Mid	Piscivore	Local	Intermediate	Nekt maneuv
	Serrasalmus maculatus	Mid	Piscivore	Local	Intermediate	Nekt maneuv
	Serrasalmus rhombeus	Mid	Piscivore	Local	Intermediate	Nekt maneuv
	Serrasalmus serrulatus	Mid	Omnivore	Local	Intermediate	Nekt maneuv
	Serrasalmus spilopleura	Mid	Piscivore	Local	Intermediate	Nekt maneuv
	Acestrorhynchidae					
Dentudo/ Peixe-cachorro	*Acestrorhynchus abbreviatus*	None	Piscivore	Local	Periodic-small	Nekt swim
	Acestrorhynchus falcatus	None	Piscivore	Local	Periodic-small	Nekt swim
	Acestrorhynchus falcirostris	None	Piscivore	Local	Periodic-small	Nekt swim
	Acestrorhynchus heterolepis	None	Piscivore	Local	Periodic-small	Nekt swim
	Acestrorhynchus nasutus	None	Piscivore	Sedentary	Periodic-small	Nekt swim

Table S1: Continued

Common name	Family<br>Species	Fishery importance	Trophic	Migratory	Life history	Habitat use
	Apteronotidae					
Sarapó	*Parapteronotus hasemani*	None	Invertivore	Local	Periodic-small	Gymnotif
	Sternarchella schotti	None	Invertivore	Local	Periodic-small	Gymnotif
	Auchenipteridae					
Mandubé	*Ageneiosus ucayalensis*	None	Piscivore	Local	Intermediate	Benthic-slow
	Ageneiosus vittatus	None	Piscivore	Local	Intermediate	Benthic-slow
	Epapterus dispilurus	None	Invertivore	Sedentary	Intermediate	Benthic-slow
	Tympanopleura atronasus	None	Piscivore	Local	Intermediate	Benthic-slow
	Tympanopleura piperata	None	Piscivore	Local	Intermediate	Benthic-slow
Mandii de boto	*Centromochlus heckelii*	None	Omnivore	Sedentary	Intermediate	Benthic-slow
Cangati	*Parauchenipterus sp. porosus*	None	Omnivore	Sedentary	Intermediate	Benthic-slow
	Trachelyopterus galeatus	None	Omnivore	Sedentary	Intermediate	Benthic-slow
	Belonidae					
Agulhão prata	*Pseudotylosurus microps*	None	Pisc./MacInv	Local	Periodic-small	Nekt swim
	Characidae					
Sardinha dentuda	*Agoniates anchovia*	None	Piscivore	Local	Periodic-small	Nekt maneuv
Piabão	*Astyanax abramis*	None	Omnivore	Local	Periodic-small	Nekt maneuv
Peixe cão	*Charax gibbosus*	None	Piscivore	Local	Periodic-small	Epib maneuv
	Charax michaeli	None	Piscivore	Local	Periodic-small	Epib maneuv
	Charax tectifer	None	Piscivore	Local	Periodic-small	Epib maneuv
Piaba	*Moenkhausia cf.megalops*	None	Omnivore	Sedentary	Opportunistic	Nekt maneuv
Zé-do-ó	*Roeboides affinis*	None	Pisc./MacInv	Sedentary	Opportunistic	Epib maneuv
	Roeboides myersii	None	Pisc./MacInv	Local	Periodic-small	Epib maneuv
-	*Stichonodon insignis*	None	Invertivore	Sedentary	Opportunistic	Nekt maneuv
Matupiri	*Tetragonopterus argenteus*	None	Omnivore	Local	Periodic-small	Epib maneuv
-	*Stethaprion erythrops*	None	Invertivore	Local	Periodic-small	Nekt maneuv
	Chilodontidae					
Cabeça-dura	*Caenotropus labyrinthicus*	None	Omnivore	Local	Periodic-small	Nekt maneuv
	Cichlidae					
Papa terra	*Acarichthys heckelii*	None	Invertivore	Sedentary	Equilibrium-small	Epib maneuv
Acará lanterna	*Acaronia nassa*	None	Pisc./MacInv	Sedentary	Equilibrium-small	Epib maneuv

Table S1: Continued

Common name	Family Species	Fishery importance	Trophic	Migratory	Life history	Habitat use
Rabo de fogo	*Chalceus macrolepidotus*	None	Invertivore	Local	Periodic-small	Nekt maneuv
Acará cascudo	*Cichlasoma amazonarum*	None	Omnivore	Sedentary	Equilibrium-small	Epib maneuv
Acará bandeira	*Pterophyllum scalare*	None	Invertivore	Sedentary	Equilibrium-small	Epib maneuv
	Ctenoluciidae					
Bicuda	*Boulengerella maculata*	None	Piscivore	Local	Periodic-large	Nekt swim
	Curimatidae					
Peito de aço	*Cyphocharax abramoides*	None	Detritivore	Local	Periodic-small	Epib maneuv
	Cyphocharax spiluropsis	None	Detritivore	Local	Periodic-small	Epib maneuv
Curimatai	*Steindachnerina bimaculata*	None	Detritivore	Local	Periodic-small	Epib maneuv
	Doradidae					
	Agamyxis pectinifrons	None	Omnivore	Sedentary	Periodic-small	Benthic-slow
	Amblydoras affinis	None	Omnivore	Sedentary	Periodic-small	Benthic-slow
	Amblydoras spinossissimus	None	Omnivore	Sedentary	Periodic-small	Benthic-slow
	Anadoras grypus	None	Omnivore	Sedentary	Periodic-small	Benthic-slow
	Nemadoras sp.	None	Omnivore	Local	Periodic-small	Benthic-fast
Reque-reque	*Opsodoras stuebelii*	None	Invertivore	Local	Periodic-small	Benthic-slow
	Ossancora punctata	None	Invertivore	Sedentary	Intermediate	Benthic-slow
	Tenellus leporhinus	None	Omnivore	Local	Periodic-small	Benthic-fast
	Tenellus ternetzi	None	Invertivore	Local	Periodic-small	Benthic-slow
	Trachydoras nattereri	None	Invertivore	Sedentary	Intermediate	Benthic-slow
	Trachydoras steindachneri	None	Invertivore	Sedentary	Intermediate	Benthic-slow
Bacui	*Platydoras costatus*	None	Omnivore	Sedentary	Periodic-small	Benthic-slow
	Engraulidae					
	Jurengraulis juruensis	None	Planktivore	Local	Periodic-small	Nekt maneuv
Sardinha	*Anchoviella guianensis*	None	Planktivore	Sedentary	Opportunistic	Nekt maneuv
	Lycengraulis batesii	None	Pisc./MacInv	Local	Periodic-small	Nekt maneuv
	Gasteropelecidae					
Sardinha papuda	*Thoracocharax securis*	None	Omnivore	Local	Periodic-small	Epib maneuv
	Gymnotidae					
Poraque	*Electrophorus electricus*	None	Piscivore	Sedentary	Equilibrium-large	Benthic-slow
Sarapó	*Gymnotus arapaima*	None	Pisc./MacInv	Sedentary	Equilibrium-small	Gymnotif
	Hemiodontidae					
Reque-reque	*Hemidoras morrisi*	None	Omnivore	Local	Periodic-small	Benthic-fast

Table S1: Continued

Common name	Family<br>Species	Fishery importance	Trophic	Migratory	Life history	Habitat use
	Loricariidae					
Cascudo/ acarizinho/ bodozinho	*Hypostomus plecostomus*	Mid	Detritivore	Sedentary	Equilibrium-large	Benthic-slow
	Hypostomus carinatus	None	Detritivore	Sedentary	Equilibrium-large	Benthic-slow
	Hypostomus oculeus	None	Detritivore	Sedentary	Equilibrium-large	Benthic-slow
	Dekeyseria amazonica	None	Detritivore	Sedentary	Equilibrium-small	Benthic-slow
	Hypoptopoma gulare	None	Detritivore	Sedentary	Intermediate	Benthic-slow
	Limatulichthys griseus	None	Omnivore	Sedentary	Equilibrium-large	Benthic-slow
Acari chato	*Loricaria cf. nickeriensis*	None	Detritivore	Sedentary	Equilibrium-small	Benthic-slow
	Loricaria sp.	None	Detritivore	Sedentary	Equilibrium-small	Benthic-slow
	Loricariichthys sp.	None	Omnivore	Sedentary	Equilibrium-large	Benthic-slow
Limpa Vidro	*Otocinclus sp.*	None	Detritivore	Sedentary	Opportunistic	Benthic-slow
Braço-de-moça	*Hemisorubim platyrhynchos*	None	Piscivore	Local	Periodic-large	Benthic-fast
Bico de pato	*Sorubim lima*	None	Piscivore	Local	Periodic-large	Benthic-fast
	Pristigasteridae					
Sardinha papuda	*Pristigaster cayana*	None	Planktivore	Local	Periodic-small	Epib maneuv
	Rhamphichthyidae					
Sarapó	*Gymnorhamphichthys sp.*	None	Invertivore	Sedentary	Periodic-small	Gymnotif
	Rhamphichthys marmoratus	None	Invertivore	Local	Periodic-large	Gymnotif
	Rhamphichthys rostratus	None	Invertivore	Local	Periodic-large	Gymnotif
	Sternopygidae					
	Eigenmannia limbata	None	invertivore	Local	Periodic-small	Gymnotif
	Sternopygus macrurus	None	Invertivore	Local	Periodic-large	Gymnotif
	Tetraodontidae					
Baiacu	*Colomesus asellus*	None	Omnivore	Sedentary	Periodic-small	Benthic-slow
